# Enhancement of dye regeneration kinetics in dichromophoric porphyrin–carbazole triphenylamine dyes influenced by more exposed radical cation orbitals†
†Electronic supplementary information (ESI) available: Transient absorption signal analysis, UV-visible absorption spectra, TD-DFT calculated cation spectra and orbitals, spectro-electrochemical spectra, transient absorption signal fitted parameters. See DOI: 10.1039/c6sc00429f


**DOI:** 10.1039/c6sc00429f

**Published:** 2016-03-01

**Authors:** Long Zhao, Pawel Wagner, Jonathan E. Barnsley, Tracey M. Clarke, Keith C. Gordon, Shogo Mori, Attila J. Mozer

**Affiliations:** a ARC Centre of Excellence for Electromaterials Science , Intelligent Polymer Research Institute , University of Wollongong , Wollongong , NSW 2522 , Australia . Email: attila@uow.edu.au ; Tel: +61 242981429; b MacDiarmid Institute for Advanced Materials and Nanotechnology , Department of Chemistry , University of Otago , Dunedin , New Zealand; c Division of Chemistry and Materials , Faculty of Textile Science and Technology , Shinshu University , Ueda , Nagano 386-8567 , Japan

## Abstract

Reduction kinetics of oxidized dyes absorbed on semiconductor surfaces and immersed in redox active electrolytes has been mainly modeled based on the free energy difference between the oxidation potential of the dye and the redox potential of the electrolyte. Only a few mechanisms have been demonstrated to enhance the kinetics by other means. In this work, the rate constant of the reduction of oxidized porphyrin dye is enhanced by attaching non-conjugated carbazole triphenylamine moiety using iodine/triiodide and tris(2,2′-bispyridinium)cobalt II/III electrolytes. These results are obtained using transient absorption spectroscopy by selectively probing the regeneration kinetics at the porphyrin radical cation and the carbazole triphenylamine radical cation absorption wavelengths. The enhancement in the reduction kinetics is not attributed to changes in the driving force, but to the more exposed dye cation radical orbitals of the dichromophoric dye. The results are important for the development of high efficiency photo-electrochemical devices with minimalized energy loss at electron transfer interfaces.

## Abstract 2

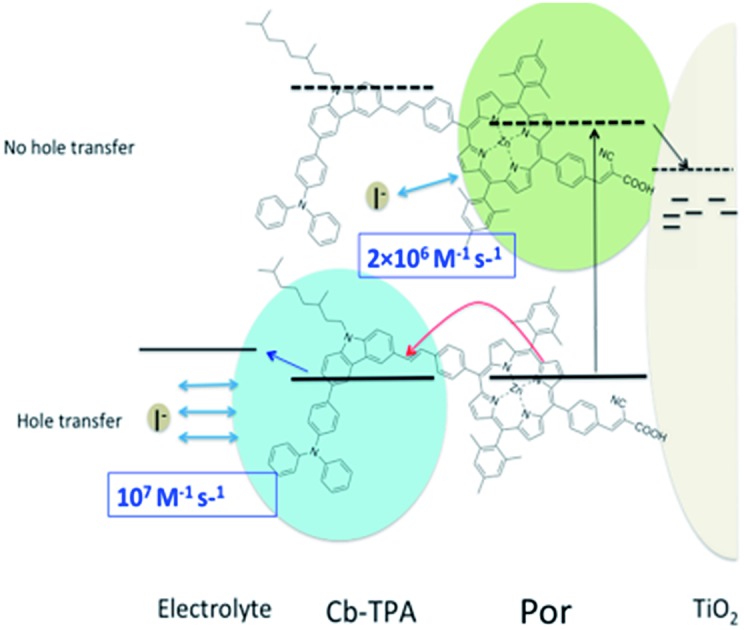
Dye regeneration kinetics is enhanced by five times in a di-chromophoric porphyrin dye by attaching tridimensionally enlarged electron donor moieties.

## Introduction

Dye-sensitization of semiconductor electrodes immersed in electrolyte solutions has been applied to various applications. To better utilize the energy of the absorbed photons, energy differences at electron transfer interfaces should be minimized. For the case of dye-sensitized solar cells (DSSCs), dyes are adsorbed on a semiconductor surface and the electrode is immersed in electrolyte solutions containing redox couples. Under light irradiation, photo-excited electrons in the dyes are injected into the conduction band of the semiconductor. The resulting dye cation radicals need to be reduced by electron donors in the electrolytes faster than by the injected electrons. To reduce the dye cation radicals efficiently, typically more than 0.4 eV free energy difference between the oxidation potential of the dye and the redox potential of the redox electrolyte is required.^1^,^2^ Several reports show efficient oxidized dye reduction with less free energy difference, but little knowledge about the mechanism for such enhanced kinetics at a reduced driving force has been obtained.^3^–^5^ As a consequence, no clear molecular design guides are available for improved sensitizers with better solar energy conversion efficiencies.

**Fig. 1:**
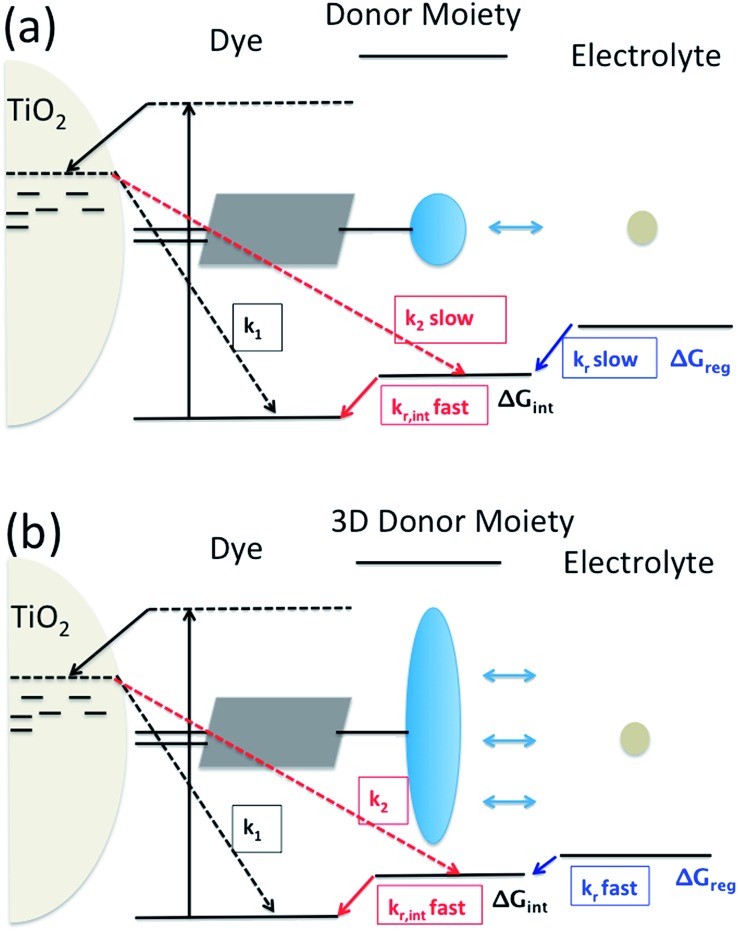
Utilization of intramolecular hole transfer to enhance dye cation regeneration. (a) Reduced recombination and slower regeneration kinetics; (b) faster dye regeneration kinetics at a smaller driving force using a tridimensional donor moiety.

It has been proposed to covalently attach an additional electron donor moiety to sensitizers so that the photo-generated hole in the sensitizer could be transferred to the donor moiety, sometimes referred to as intramolecular charge regeneration (Fig. 1).^6^ The rate of hole transfer can be changed by controlling the electronic coupling and the driving force for hole transfer between the units.^7^–^9^ Since the distance between the injected electrons and holes in the electron donor modified – dye molecules can be increased, recombination between the electron and the hole can be retarded (*k*_2_ slower than *k*_1_). The longer lifetime of the charge separated state allows slower dye cation reduction kinetics, in principle enabling less free energy difference (Δ*G*_reg_). This mechanism of enhancement is illustrated in Fig. 1(a).

However, so far no successful reports demonstrating enhanced reduction kinetics due to internal hole transfer have been published. This could be because a large, additional energy difference between the sensitizer and the new moiety (Δ*G*_int_) to drive efficient hole transfer is typically introduced. If the same redox mediator is used, the reduction kinetics of the oxidized electron donor moiety may be too slow due to too small Δ*G*_reg_ between the electron donor moiety and the redox mediator in the electrolyte (Fig. 1(a)).

Another pathway to utilize the internal hole transfer mechanism is to increase the regeneration kinetics by attaching electron donor moieties with faster regeneration kinetics at small driving forces to sensitizers (Fig. 1(b)). By attaching donor units with increased tridimensionality, the exposed area of the oxidized dye orbitals to the electrolyte could be enlarged, leading to enhanced collision cross section between the electron donor in the electrolyte and the dye. Porphyrin dyes having a planar structure, typically oriented perpendicular to the semiconductor surface due to the geometry of the binding site, are good candidates to test the merit of this novel mechanism, which is the aim of this work.

**Fig. 2:**
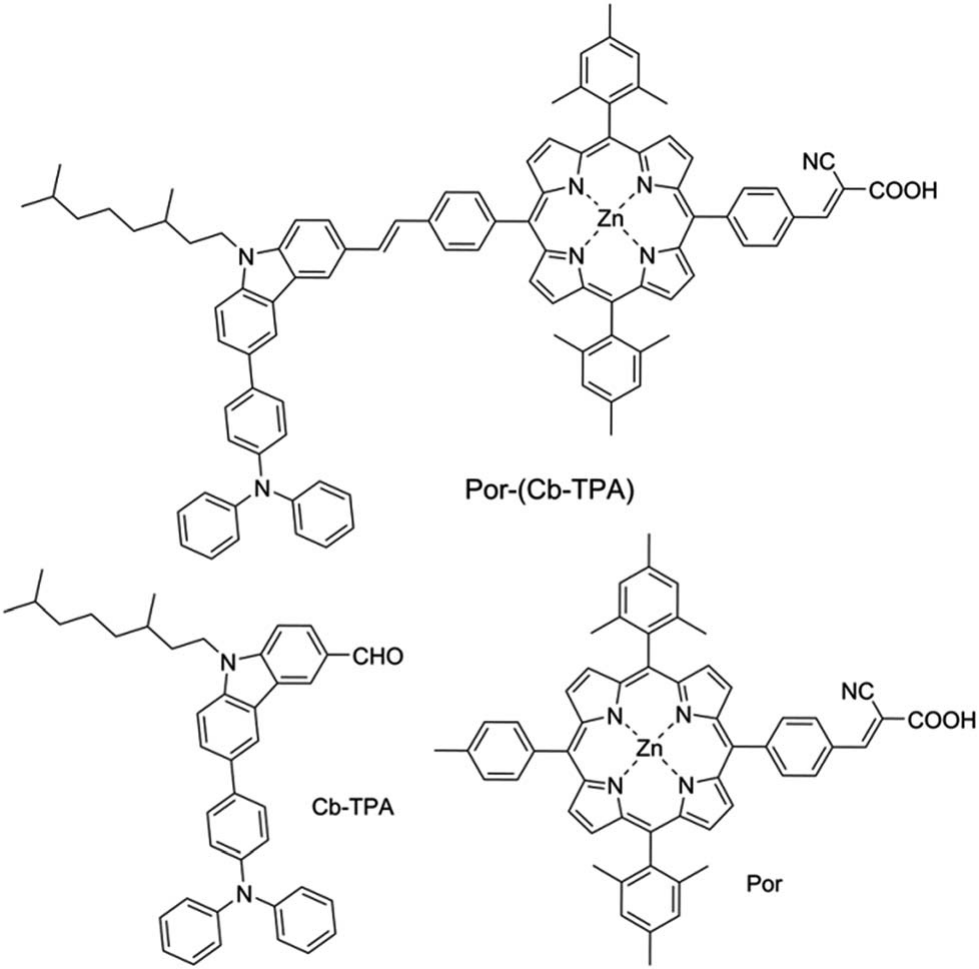
Chemical structures of the compounds.

We have developed a series of dichromophoric porphyrin dyes based on porphyrin and carbazole chromophores linked by a phenylethenyl linker.^10^Fig. 2 shows a recent example of a dichromophoric dye 5-(4-(2-cyano-2-carboxyethenyl)phenyl-15-(4-(2-(6-(4-(*N*,*N*-diphenylamino))))phenyl-9-(3,7-dimethyloctyl)-carbazol-3-yl)ethenyl)phenyl-10,20-bis(2,4,6-trimethylphenyl)porphyrinato zinc(ii) (Por–(Cb-TPA)) consisting of a porphyrin 5-(4-(2-cyano-2-carboxyethenyl))phenyl-15-(4-methylphenyl)-10,20-bis(2,4,6-trimethylphenyl)porphyrinato zinc(ii) (Por) and 6-(4-(*N*,*N*-diphenylamino)phenyl)-(9-(3,5-dimethyloctyl))carbazole-3-carboxaldehyde (Cb-TPA) chromophores. The dihedral twist of 117 degrees minimized electronic communication between the two chromophores. This non-conjugated design generally leads to higher extinction coefficients and/or panchromatic absorption spectrum, without the appearance of new absorption bands in the dichromophoric dye (Fig. S1†).^11^ The dichromophoric dye Por–(Cb-TPA) when used in dye-sensitized solar cells showed improved photovoltaic performance compared to Por, attributed to improved electron injection and reduced TiO_2_ conduction band electron recombination with oxidized species in the electrolyte.^10^ Both of these improvements could be traced back to the more bulky molecular structure of Por–(Cb-TPA). On one hand, the improved dye morphology on the TiO_2_ surface eliminated the use of a coadsorber to control dye aggregation. On the other hand, the blocking effect of the bulky substituent of the dye prevented the approach of the oxidized species in the electrolyte to the TiO_2_ surface blocking charge recombination. An overall 30% improvement compared to the Por dye in DSSCs was demonstrated.^10^

Here, we show that the attachment of a Cb-TPA electron donor moiety to Por dye enhances the dye radical cation reduction kinetics in Por–(Cb-TPA) as compared to the Por dye. The oxidation potentials of the Por and Cb-TPA chromophores were found to be within 0.1 V of each other, leading to insignificant changes in the free energy difference for regeneration. Due to this, the hole transfer, observed by selectively probing the oxidized cation radical absorption changes using transient absorption spectroscopy, is slow and partial. The enhanced dye cation radical regeneration of the dichromophoric dye Por–(Cb-TPA) is interpreted with larger exposed dye cation radical orbitals of the Por–(Cb-TPA) to the electrolyte solution so that redox mediator in electrolyte can interact more easily with the dye radical cation orbitals. This work, although does not lead to practically significant benefits to DSSC performance, demonstrates a new method to increase the dye cation reduction kinetics without increasing the free energy difference.

## Experimental section

### Materials

Dichloromethane (DCM, 99.8%, Chem-Supply), tetrabutylammonium perchlorate (TBAP, 99.0%, Fluka), ferrocene (Fc, 98%, Aldrich), titanium diisopropoxide bis(acetylacetonate) (TAA, 75% in isopropanol, Aldrich), ethanol (99.8%, Sigma-Aldrich), TiO_2_ paste (18-NRT, Dyesol), titanium IV chloride (TiCl_4_, 99.0%, Sigma-Aldrich), tetrahydrofuran (THF, 97%, Ajax), 1,2-dimethyl-3-propylimidazolium iodide (DMPII, synthesized in house), lithium iodide (LiI, 99.9%, Aldrich), I_2_ (99.99%, Aldrich), *tert*-butylpyridine (*t*BP, 96%, Aldrich), acetonitrile (AN, 99.8%, Sigma-Aldrich), valeronitrile (VN, 99.5%, Sigma-Aldrich), tris(2,2′-bispyridine)cobalt II/III (Co^2+^/Co^3+^, synthesized in house), chenodeoxycholic acid (CDCA, Solaronix), fluorine doped tin oxide glass (FTO glass, 2.2 mm, 7 Ω □^–1^, TEC®), glass microscope slides (Sail brand, 1 mm thick).

Por–(Cb-TPA), Cb-TPA and Por were synthesized as described in ref. 10.

### DFT energy calculations

DFT energy calculations were generated using the Gaussian 09W program package which implemented the Becke, three-parameter, Lee–Yang–Parr (B3LYP) model employing the basis set 6-31G(d).^12^ A ground state Por–(Cb-TPA) structure was optimized and was found to lie in an energy minimum due to the absence of imaginary frequencies. A copy of this structure was fragmented to form Por, Cb, (Cb-TPA) and TPA moieties which were oxidized and re-optimized to form Por^+^, Cb^+^, (Cb-TPA)^+^ and TPA^+^ radical structures respectively. The ground state structure was augmented with these oxidized radical structures to form Por^+^–Cb-TPA, Por–Cb^+^-TPA, Por–(Cb-TPA)^+^ and Por–Cb-TPA^+^ structures with identical bond length, angles and torsion angles to the ground state geometry. Single point energy calculations in vacuum for each of these species were carried out to give relative energies for each species. The calculation approach for the oxidized species is shown schematically in the ESI Scheme S1.† The visualization of molecular orbitals was provided using Gauss View 5.0 W (Gaussian Inc.).^12^

### Differential pulse voltammetry (DPV) measurement

Differential Pulse Voltammetry (DPV) measurement was carried out in DCM with a three-electrode system using a potentiostat (eDAQ instrument). The solvent was dried by going through a column of activated alumina. Prior to each experiment, the solvent was purged with dry argon for 30 minutes, and placed in a glove bag (Atmosbag, Sigma-Aldrich). The concentration of the dissolved dyes was 0.5 mM and the concentration of TBAP, used as supporting electrolyte, was 0.1 M. DPV measurements were performed using a Pt wire with 0.5 mm diameter as the working electrode (WE), Pt mesh as the counter electrode (CE) and a Ag/AgCl wire as the pseudo reference electrode (RE). The RE potential was calibrated using 1 mM ferrocene dissolved in the same solution as the dye.

### Spectro-electrochemical (SEC) spectroscopy

Spectro-electrochemical (SEC) spectroscopy was performed by monitoring the change in the light absorption (Shimadzu UV-3600 spectrometer) of the dyes in solution during electrochemical oxidation under steady state applied potentials. An optically transparent, thin-layer (path length around 0.2 mm) three-electrode electrochemical (OTTLE) cell was used, with a platinum mesh as the working and counter electrodes, and a silver wire as the reference electrode. Applied potentials were calibrated by cyclic voltammetry measurements of 1 mM Fc/Fc^+^ redox electrolyte in the same electrochemical cell. The dyes were dissolved in DCM (0.2 mM) together with 0.1 M TBAP as supporting electrolyte. First, the absorption spectrum of the neutral dyes were recorded at –0.2 V *vs.* Fc/Fc^+^. This baseline spectrum was subtracted from the absorption spectra recorded at other potentials resulting in the data represented as the absorption difference at each applied potential (ΔAbs). 2–5 minutes passed between applying a potential step and taking the absorption spectrum allowing the current to decrease to close to zero.

### Sample fabrication

Dye-sensitized TiO_2_ electrodes consisted of a compact TiO_2_ and a mesoporous TiO_2_ layer. The former was deposited by spray coating of a TAA solution diluted by ethanol with a volume ratio of 1 : 9 onto a cleaned FTO glass held at 400 °C and then sintered for 30 minutes. The mesoporous TiO_2_ layer was around 3 μm thick, and the geometric area of the electrode was 8 mm × 8 mm, deposited by screen printing of a transparent TiO_2_ paste and sintered following the procedure: 325 °C for 5 minutes, 375 °C for 5 minutes, 450 °C for 30 minutes and 500 °C for 15 minutes. The mesoporous layer was immersed into 0.02 M TiCl_4_ solution in water for post-treatment by holding at 70 °C for 30 minutes. The electrodes were sintered again at 500 °C for 30 minutes. The completed TiO_2_ electrodes were placed in a dye solution: for “full dye loading”, 0.2 mM dye in THF for 90 minutes; for “reduced dye loading” of Por–(Cb-TPA), 0.05 mM dye in THF for 90 minutes; for “reduced dye loading” of Por, 0.01 mM dye in THF for 90 minutes. The dye sensitized TiO_2_ electrodes were covered by a piece of 1 mm thick microscope glass with a pre-drilled hole at one corner, and sandwiched with a 25 μm Surlyn® film at 120 °C for 30 seconds. The electrolyte was then backfilled using a vacuum pump. The electrolyte composition is listed in Table 1. The pre-drilled hole on the microscope glass was sealed using a piece of Surlyn® attached aluminum foil.

**Table 1:** Electrolytes nomination and components

Electrolyte	Components and concentration
I_1	0.6 M DMPII, 0.1 M LiI and 50 mM I_2_ in I_0
I_0.1	0.06 M DMPII, 0.01 M LiI and 5 mM I_2_ in I_0
I_0.01	0.006 M DMPII, 0.001 M LiI and 0.5 mM I_2_ in I_0
I_0.001	0.0006 M DMPII, 0.0001 M LiI and 0.05 mM I_2_ in I_0
I_0	0.1 M LiClO_4_, and 0.5 M *t*BP in AN : VN = 85 : 15 (vol)
Co_1	0.11 M Co^2+^ and 25 mM Co^3+^ in Co_0
Co_0.1	0.011 M Co^2+^ and 2.5 mM Co^3+^ in Co_0
Co_0.01	0.0011 M Co^2+^ and 0.25 mM Co^3+^ in Co_0
Co_0.001	0.00011 M Co^2+^ and 0.025 mM Co^3+^ in Co_0
Co_0	0.05 M LiClO_4_, and 0.1 M *t*BP in AN

### Transient absorption spectroscopy (TAS)

Transient absorption spectroscopy (TAS) was performed in transmission mode using a Q-switched Nd-YAG laser (8 ns, 532 nm, 10 Hz, Quanta-Ray® INDI Spectra-physics) and a white quartz halogen lamp (BENTHAM IL1) as a probe. A combination of filters, including band, long pass and neutral density filters were used in front of the sample to adjust the probe intensity and limit the photo-excitation of the sample by the probe beam. The probe beam transmitted by the sample was dispersed by a monochromator (CM110, SP) and detected by a silicon (HCA-S-200M-Si, Femto) or an InGaAs photoreceiver (HCA-S-200M-IN, Femto). For TA spectrum measurements, the signals were further amplified by a 200 MHz voltage amplifier (DHPVA-200, Femto, AC mode with 10 Hz low frequency cut-off) with a pulse-to-pulse repetition rate of 10 Hz. The signals for kinetic measurements were not amplified and the pulse to pulse repetition rate was 1 Hz. A digital oscilloscope (DPO 4054, Tektronix) was used to digitize the voltage signals using DC coupling and 50 Ω impedance, typically averaging 512 pulses per measurement. The time resolution of the set up was around 40 ns.

To achieve nanosecond time resolution (Fig. S10†), a 100 ps laser (SL230, Ekspla®, 532 nm) was employed as excitation light source. The probe beam was a pulsed halogen lamp (Xe900, Edinburgh Instruments). The probe beam transmitted by the sample was dispersed by a monochromator (Newport) and detected by a silicon or an InGaAs photoreceiver (1601 & 1611, New focus). The measurement was performed at 1 Hz repetition rate taking 80 to 200 averages. The transients were recorded using a 2 GHz digital phosphor oscilloscope (DPO 5204, Tektronix).

### Stretched exponential decay functions

Stretched exponential decay functions were used to fit the transient absorption curves following the procedure described in the literature^4^,^13^ and explained in detail in the ESI.†

## Results

### Energetics of the oxidised cation radical in the porphyrin–carbazole triphenylamine dye

**Fig. 3:**
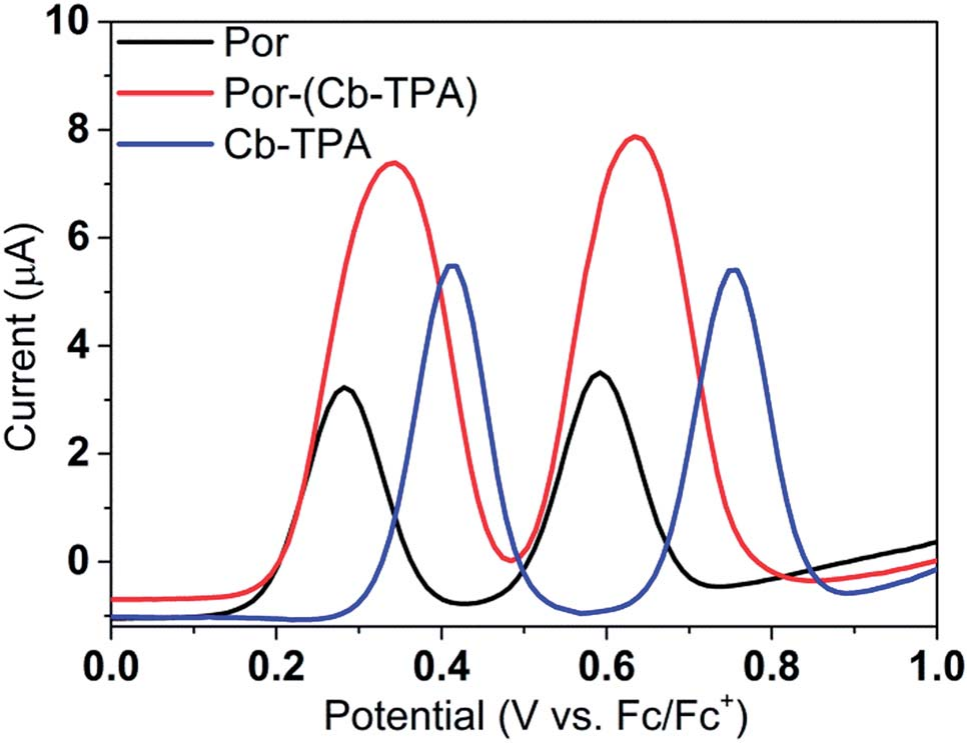
(a) Differential pulse voltammetry of the compounds measured in dichloromethane solutions.

Fig. 3 shows differential pulse voltammetry plots obtained for the porphyrin dye (Por), the carbazole-triphenylamine chromophore (Cb-TPA) and the dichromophoric dye (Por–(Cb-TPA)). The first oxidation peak of Por–(Cb-TPA) appears to be the superposition of the oxidation peaks of the constituting chromophores Por and Cb-TPA with an increased peak area, but without noticeable changes in the onset of oxidation. These results suggest negligible electronic interaction between the chromophores in the ground state, which is also supported by UV-vis absorption (Fig. S1†), DFT calculations and resonant Raman experiments.^10^ The first oxidation peak of Por–(Cb-TPA) is attributed to two one-electron oxidation reactions of the two electroactive chromophores: Por oxidised to Por^+^ and Cb-TPA oxidized to (Cb-TPA)^+^. The onset of the second oxidation peak of Por–(Cb-TPA) matches the onset of the second oxidation peak of Por. The second oxidation peak of Cb-TPA (Cb-TPA)^+^ to (Cb-TPA)^2+^ is shifted to lower potentials in Por–(Cb-TPA) from 0.75 V to 0.6 V *vs.* Fc/Fc^+^.

DFT calculated radical species were implemented to develop understanding of where the first oxidation occurs in this dye. Augmentation of the ground state structure with optimized racial species allows the generation of localized radical structures, specifically located on Por, Cb, Cb-TPA or TPA moieties (see Scheme S1†). Electron density diagrams shown in Table 2 display occupied and unoccupied orbitals (α and β SOMO locales) for the various radical species.

**Table 2:** B3LYP calculated SOMO orbitals for the various localized radical species including the energies for these species, relative to Por–(Cb-TPA)^+^

	Occupied SOMO	Unoccupied SOMO
Por–Cb^+^-TPA +0.048 eV	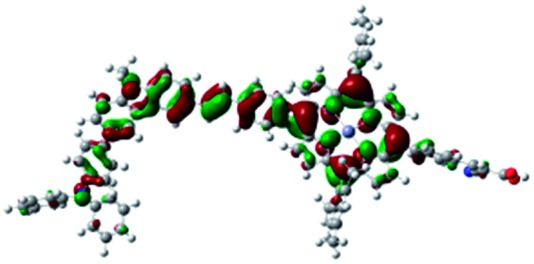	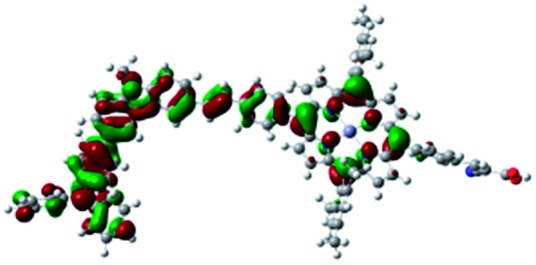
Por–(Cb-TPA)^+^ +0.012 eV	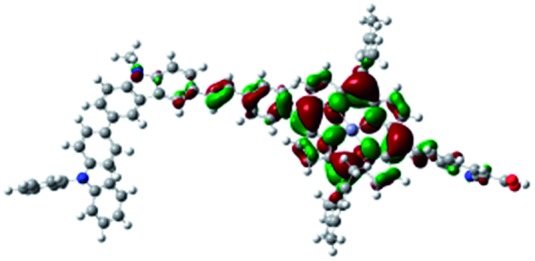	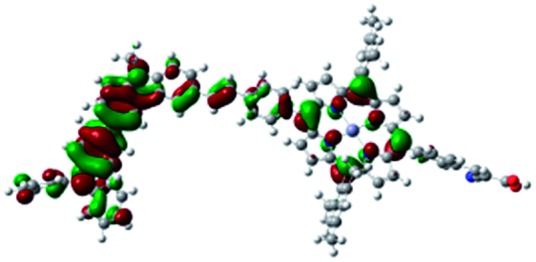
Por^+^–(Cb-TPA) +0.006	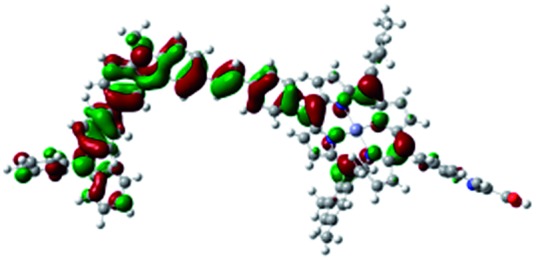	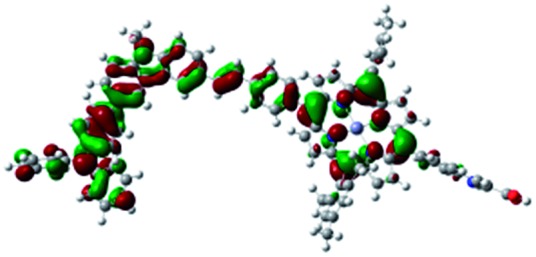
Por–(Cb-TPA)^+^	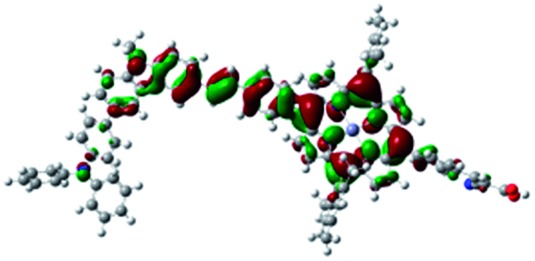	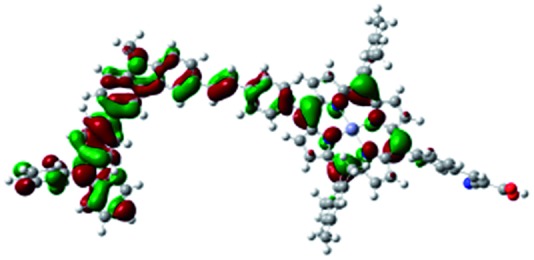

Single point energy calculations show the Por–(Cb-TPA)^+^ radical species to be the lowest in energy followed by Por^+^–(Cb-TPA) (+0.006 eV), Por–(Cb-TPA)^+^ (+0.012 eV) then Por–Cb^+^-TPA (+0.048 eV). Thermal energy at 293 K is sufficient to result in mix of these predicted states, which is consistent with differential pulse voltammetry results. The optical transitions of the oxidized radical species were calculated using TD-DFT and are displayed as oscillator strength *versus* wavelength in Fig. S2.†

### Electrochemically generated dye radical cations in the porphyrin–carbazole triphenylamine dye

Cb-TPA absorbs photons with wavelengths shorter than 400 nm. The absorption spectrum of Por–(Cb-TPA) appears to be a superposition of the absorption spectra of the constituting chromophores (ref. 10 and Fig. S1†). Therefore, at 532 nm pump wavelength, the Por chromophore is selectively photo-excited. To determine the photo-generated cation radical absorption, first, spectro-electrochemical measurements were performed.

**Fig. 4:**
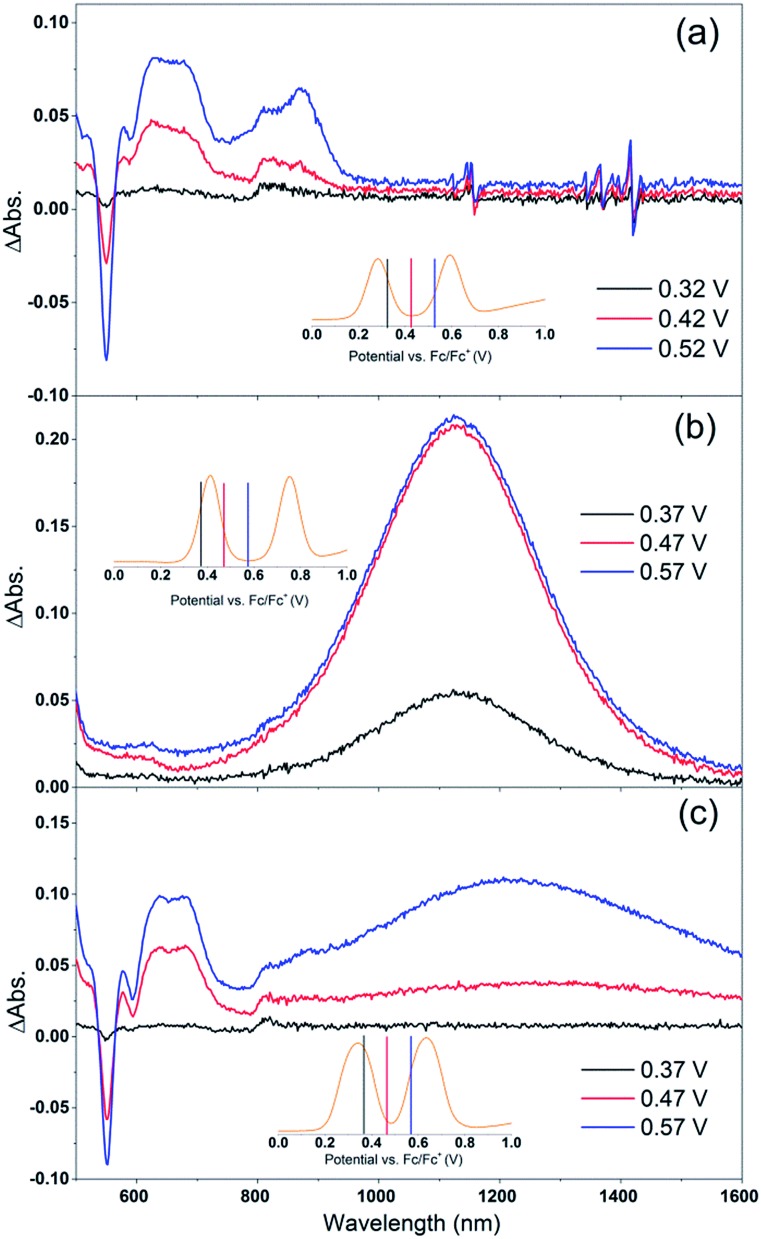
Spectro-electrochemical (SEC) spectra of (a) Por, (b) Cb-TPA and (c) Por–(Cb-TPA) at different oxidation potentials *vs.* Fc/Fc^+^. For wavelength range from 230 nm, see Fig. S4.†

Fig. 4 shows the change in the UV/Vis/NIR absorption (ΔAbs) when Por, Cb-TPA and Por–(Cb-TPA) are oxidized at various constant potentials applied referenced to the redox potential of the Fc/Fc^+^ couple. Spectra obtained at three applied potentials spanning the first oxidation peak observed in the DPV are shown. For spectra recorded at higher oxidation potentials within the second oxidation peak in the DPV plots, see Fig. S4.† The steady state potentials are illustrated as vertical lines in the DPV plots displayed in the Fig. 4 insets. The oxidation of the porphyrin (Por to Por^+^, Fig. 4(a)) yields the porphyrin cation absorption spectrum from 600 nm to 950 nm and a bleached Q band absorption from 570 nm to 600 nm. A wider spectral region from 250 nm to 1600 nm is shown in Fig. S4† displaying the bleach of the Soret band and some additional absorption features.

One electron oxidation of the Cb-TPA (Fig. 4(b)) leads to a strong ΔAbs feature centered around 1150 nm, with lower ΔAbs values found between 600 to 800 nm. The neutral Cb-TPA absorption bleach is observed below 400 nm (Fig. S4†).

Oxidation of Por–(Cb-TPA) (Fig. 4(c)) at 0.37 V Fc/Fc^+^ corresponding to the onset of oxidation in the DPV plots (Fig. 3) shows the bleaching of the porphyrin Q band absorption between 570 nm and 600 nm, and positive ΔAbs between 600 nm and 900 nm. This spectrum is similar to Por oxidation to Por^+^ (see Fig. 4(a)), suggesting that at the onset oxidation potential of the dichromophoric dye, the dominant reaction product is Por^+^–Cb-TPA. At higher potentials of 0.47 V (red line), but still within the first oxidation peak in the DPV plot, positive ΔAbs features between 1000 nm and 1600 nm, and a bleach signal below 400 nm (Fig. S4†) appears. This suggests that the oxidation product is a mixture of Por^+^–(Cb-TPA) and Por–(Cb-TPA)^+^, confirming the assignment of the first broad oxidation peak of Por–(Cb-TPA) as the superposition of two one-electron oxidation reactions of the Por and Cb-TPA chromophores.

The spectro-electrochemical data suggest that the formation of (Cb-TPA)^+^ can be selectively probed between 1000 nm to 1600 nm, and the formation of Por^+^ can be probed, albeit without perfect selectivity, between 600 nm and 900 nm.

### Dye cation radical formation and intramolecular hole transfer in the porphyrin–carbazole triphenylamine dye

**Fig. 5:**
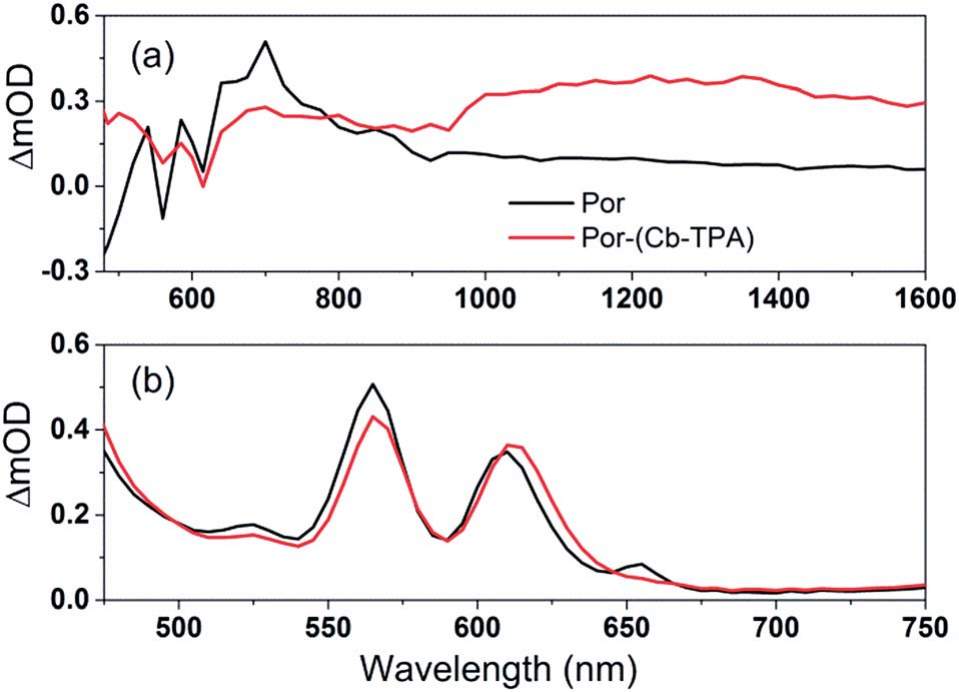
(a) Transient absorption spectra of TiO_2_–Por and TiO_2_–Por–(Cb-TPA) in the presence of the inert electrolyte I_0 at 10 μs time delay following laser excitation (532 nm, 24–26 μJ cm^–2^ per pulse) and (b) absorbance of TiO_2_–Por and TiO_2_–Por–(Cb-TPA).

Por and Por–(Cb-TPA) dyes were adsorbed on a mesoporous TiO_2_ electrode, immersed in an electrolyte containing 0.1 M LiClO_4_, and 0.5 M *t*BP in acetonitrile : valeronitrile 85 : 15 by volume electrolyte solution and sealed with a microscope cover glass. The samples were photo-excited at 532 nm with nanosecond laser pulses and changes in the transmission of the samples were recorded in the change in optical density (ΔOD) units in Fig. 5.

The transient absorption spectrum recorded at 10 μs time delay following photo-excitation of TiO_2_–Por (Fig. 5(a)) closely resembles the observed changes in the absorption following electrochemical oxidation of the compound in solution (Fig. 4(a)), with characteristic Por^+^ absorption bands between 600 nm to 900 nm and bleaching of the Q bands (superimposed on weak absorption features) below 600 nm (Fig. 5(b)). Note that the bandwidth of the TA spectra measurements was much larger (approximately 5 nm) by opening the monochromator slits to obtain better signal to noise ratio. Therefore, the TA spectra features are not as sharp as obtained by spectro-electrochemical measurements. The comparatively lower intensity and featureless transient absorption spectrum in the 900 nm to 1600 nm wavelength region is assigned to absorption by trapped electrons in the TiO_2_ (TiO_2_(e^–^)).^13^,^14^ These features and the high photon to electron conversion efficiency of the Por–TiO_2_ electrode in DSSCs^10^ suggest that photo-excitation of Por leads to the formation of TiO_2_(e^–^)–Por^+^ with high quantum yield.

The transient absorption spectrum following photo-excitation of TiO_2_–Por–(Cb-TPA) (Fig. 5(a)) shows absorption features attributed to both Por^+^ (600 nm to 900 nm) and (Cb-TPA)^+^ (1000 nm 1600 nm). The latter feature is broadened and shifted towards longer wavelengths compared to the spectrum obtained by the electrochemical oxidation of Por–(Cb-TPA) in solution (Fig. 4(c)). Transient absorption features assigned to both TiO_2_(e^–^)–Por^+^–(Cb-TPA) and TiO_2_(e^–^)–Por–(Cb-TPA)^+^ are observed, suggesting that selective photo-excitation (532 nm) of the Por chromophore in the dichromophoric dye attached to TiO_2_ leads to the photo-generation of both cation radicals.

**Fig. 6:**
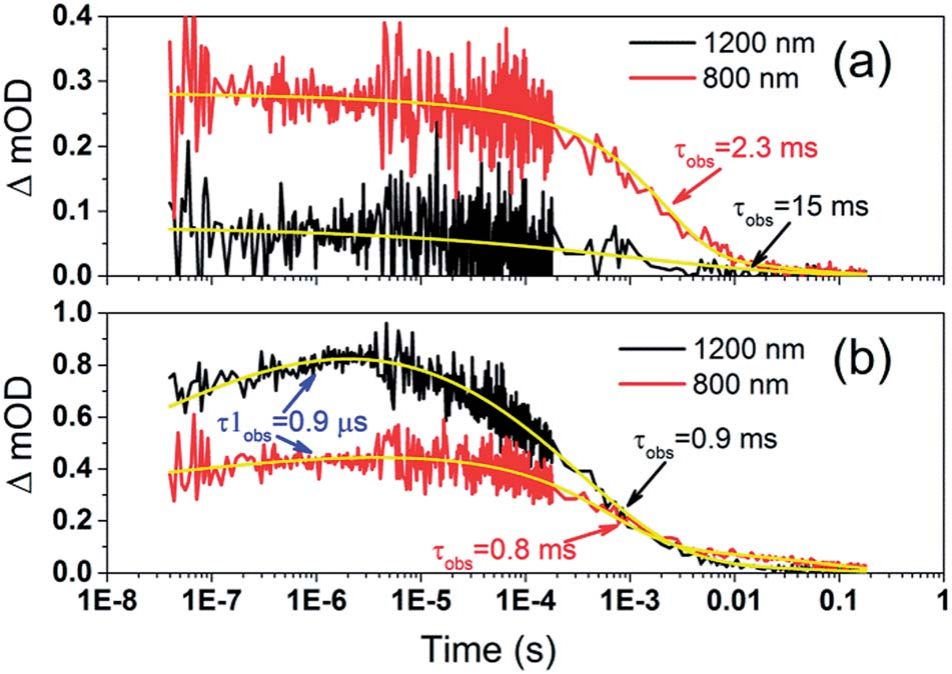
Transient absorption decays and fitted functions of (a) TiO_2_–Por and (b) TiO_2_–Por–(Cb-TPA) with the inert electrolyte I_0 (532 nm, 45–50 μJ cm^–2^ per pulse; repetition rate 1 Hz).

To examine the kinetics of the (Cb-TPA)^+^ formation in photo-excited TiO_2_–Por–(Cb-TPA), the transient absorption signal was recorded at 1200 nm and 800 nm (Fig. 6(b)). Compared to TiO_2_–Por (Fig. 6(a)) a prominent, 20% rise in the signal peaking at 10^–6^ s is observed at 1200 nm. This suggests a slow component of an intramolecular hole transfer (TiO_2_(e^–^)–Por^+^–(Cb-TPA) to TiO_2_(e^–^)–Por–(Cb-TPA)^+^). The signal at 800 nm also increases on the same timescale, although much less prominently due to the lower contribution of the absorption by Cb-TPA^+^ and decreasing signal of Por^+^ at this wavelength (see Fig. 4(b) and (c)). Bleaching signals are not shown, due to the strong possibility of sample damage during the direct photo-excitation by the probe beam in the absence of the redox mediator. For all measurements, a combination of long pass and band pass filters were used to prevent the direct photo-excitation of the dye as well as limiting electron accumulation in the TiO_2_ by the probe beam.

Intramolecular hole transfer from the Por^+^ to Cb-TPA leads to somewhat shorter dye cation radical lifetime indicated by the observed lifetime of 2.3 ms at 800 nm for TiO_2_(e^–^)–Por^+^ (Fig. 6(a)) and observed lifetime of 0.9 ms at 800 nm for TiO_2_(e^–^)–Por–(Cb-TPA)^+^ (Fig. 6(b)).

The recombination lifetimes probed at 800 nm and at 1200 nm in TiO_2_(e^–^)–Por–(Cb-TPA)^+^ are very similar, while the signal at 800 nm showed longer residual tail. The observed lifetimes obtained from fitting to a stretched exponential function suggest an average lifetime for a highly heterogeneous process.^13^ To examine this more closely, the decays at 800 nm were normalized to the same initial magnitude and plotted on the linear scale in Fig. S5,† showing longer lived TiO_2_(e^–^)–Por^+^–(Cb-TPA) in comparison to both TiO_2_(e^–^)–Por^+^ and TiO_2_(e^–^)–Por–(Cb-TPA)^+^.

### Regeneration kinetics of dye radical cations in the porphyrin–carbazole triphenylamine dye

Por and Por–(Cb-TPA) sensitized TiO_2_ electrodes were immersed in the electrolyte as above, but with added redox mediators iodide/triiodide and tris(2,2′-bispyridinium)cobalt II/III. The highest concentration of I^–^/I_3_^–^ concentration was 0.65 M/0.05 M (calculated assuming that the I_3_^–^ concentration equals the I_2_ concentration added to the electrolyte), and the highest concentration of Co^2+^/Co^3+^ was 0.11 M/0.025 M. The concentration of the redox mediators in both electrolytes was lowered by three orders of magnitude in three equal steps to check the regeneration rate constant.

**Fig. 7:**
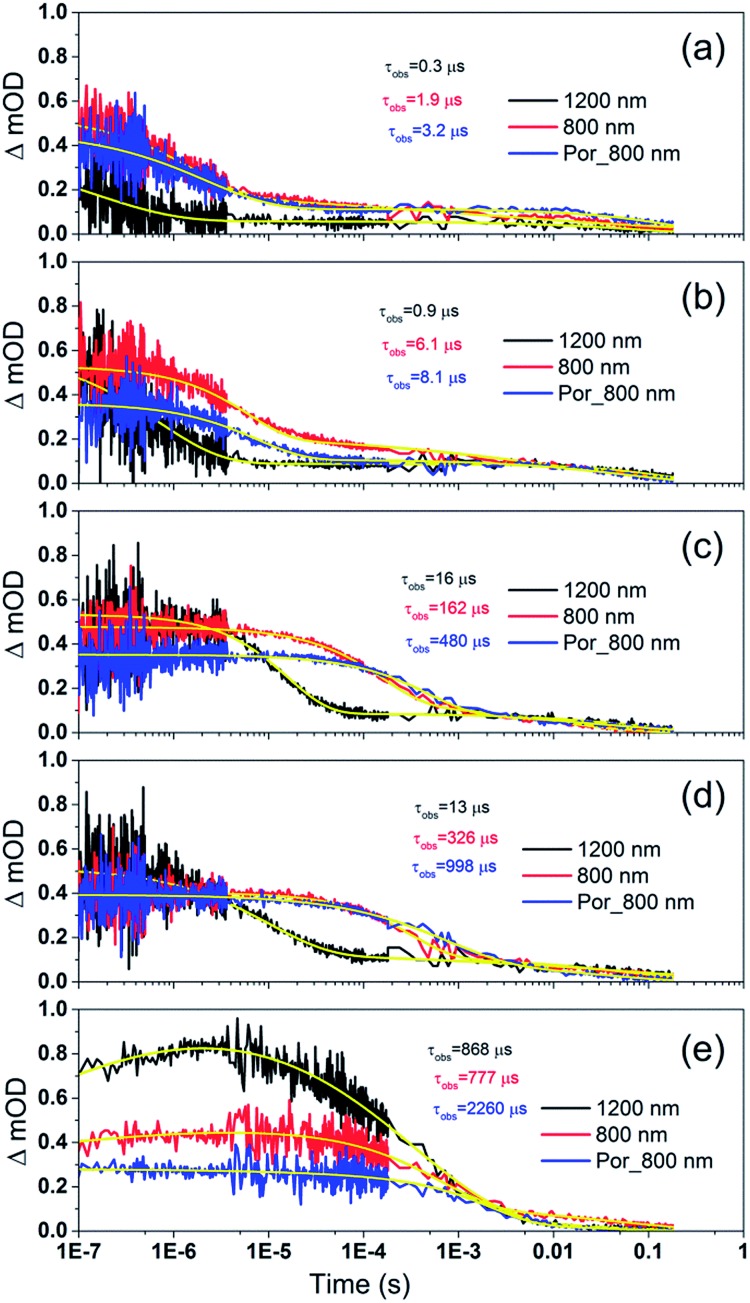
Transient absorption decay and fitted curves of TiO_2_–Por–(Cb-TPA) probed at 1200 nm (black) and 800 nm (red) and TiO_2_–Por probed at 800 nm (blue) with five I^–^/I_3_^–^ electrolytes after pulsed 532 nm laser irradiation. (a) I_1, (b) I_0.1, (c) I_0.01, (d) I_0.001 and (e) I_0 (532 nm, 45–50 μJ cm^–2^ per pulse; repetition rate 1 Hz).

Fig. 7 shows the transient absorption signal decays of photo-excited TiO_2_–Por–(Cb-TPA) and TiO_2_–Por recorded in the presence of I^–^/I_3_^–^ electrolytes at different redox concentrations. At the highest redox concentration (Fig. 7(a)), a fast decay at 800 nm is observed due to fast regeneration. Lower concentration of the redox mediator in electrolyte results in longer *τ*_obs_ indicating slower regeneration kinetics.

At all concentrations, transient absorption signal decays at 1200 nm (Por–(Cb-TPA)^+^ selectively probed, black lines) decay faster than at 800 nm (red lines). This suggests that the reduction of Por–(Cb-TPA)^+^ is faster than the reduction of Por^+^–(Cb-TPA) using iodide as electron donor. The signal decays measured at 800 nm for TiO_2_(e^–^)–Por^+^ (blue) and TiO_2_(e^–^)–Por^+^–(Cb-TPA) is more similar, with the latter being by a factor of two to three faster depending on the electrolyte concentration used.

Similar set of decay curves with a similar trend of faster decays at 1200 nm compared to 800 nm has been observed using the Co^2+^/Co^3+^ mediator (Fig. S6†), suggesting that the observed faster reduction kinetics is not specific to iodide electrolytes.

To quantitatively describe the above results, the transient absorption decay curves were fitted to a stretched exponential function (see ESI eqn S1–S4†). For comparison, signal decay half times (*t*_1/2_) have also been calculated and shown in Fig. S7 and S8.† Dye cation radical regeneration rate constants were calculated assuming a kinetic competition between dye cation radical recombination with TiO_2_ electrons (inert electrolyte) and the decay kinetics in the presence of the mediator using eqn S5 (ESI†).

**Fig. 8:**
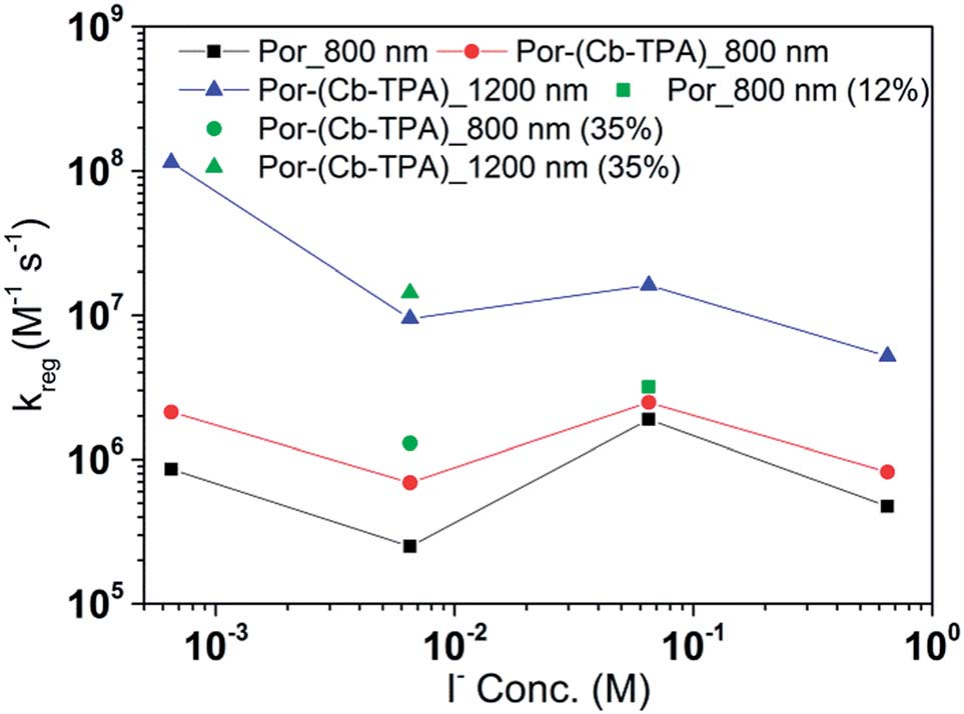
Regeneration rate constant of Por and Por–(Cb-TPA) *versus* the concentration of I^–^. Por with 12% dye-loading probed at 800 nm and Por–(Cb-TPA) with 35% dye-loading probed at 800 nm and 1200 nm are also shown.

**Fig. 9:**
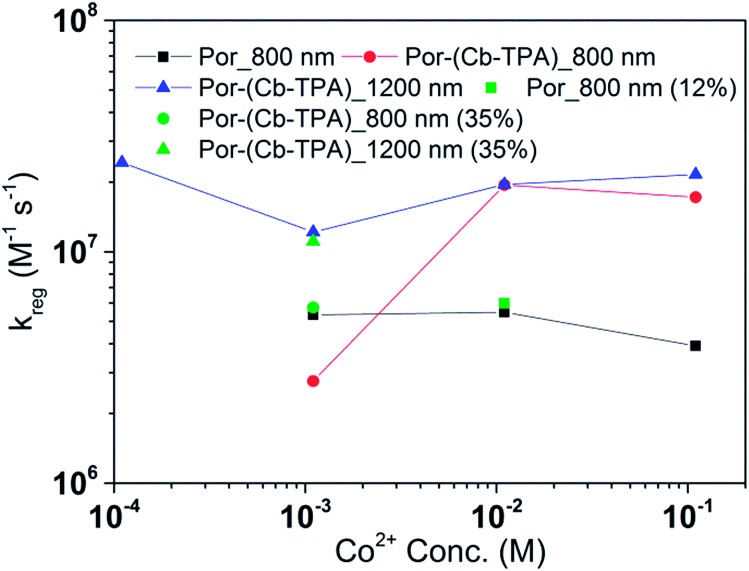
Regeneration rate constant of Por and Por–(Cb-TPA) *versus* the concentration of Co^2+^. Por with 12% dye-loading probed at 800 nm and Por–(Cb-TPA) with 35% dye-loading probed at 800 nm and 1200 nm are also shown.

Fig. 8 and 9 compare the radical dye cation TiO_2_(e^–^)–Por^+^, TiO_2_(e^–^)–Por^+^–Cb-TPA (probed at 800 nm) and TiO_2_(e^–^)–Por–(Cb-TPA)^+^ (probed at 1200 nm) regeneration time constants at various concentrations of the redox mediator I^–^/I_3_^–^ and Co^2+^/Co^3+^, respectively. Showing the rate constant *versus* concentration is chosen as any deviation from linearity at low or high concentrations can be readily observed. Alternatively, the observed rate is shown *versus* the concentration in Fig. S9† with calculated reaction order and rate constant obtained by linear fit of the data.

The calculated regeneration rate constant of Por–(Cb-TPA)^+^ was approximately 10^7^ M^–1^ s^–1^ for concentrations 0.65 to 0.65 × 10^–2^ M I^–^, which was five times faster than the regeneration rate constant of Por^+^–(Cb-TPA). The regeneration rate constant of Por^+^–(Cb-TPA) (averaged rate constant approximately 2 × 10^6^ M^–1^ s^–1^) was by a factor of two faster than the regeneration rate constant of Por^+^ (approximately 0.8 × 10^6^ M^–1^ s^–1^). The regeneration rate contant of Por–(Cb-TPA)^+^ was an order of magnitude faster (10^–8^ to 10^6^ M^–1^ s^–1^) at the lowest concentration of the mediator used.

The regeneration rate constant of Por–(Cb-TPA)^+^ by Co^2+^ was similar to its regeneration rate constant by I^–^ (approximately 10^7^ M^–1^ s^–1^). Moreover, the regeneration rate contant of Por–(Cb-TPA)^+^ was five times faster than the regeneration rate constant of Por^+^ at all Co^2+^ concentrations. The regeneration rate contant of Por^+^–(Cb-TPA), on the other hand, was similar to the regeneration rate constant of Por–(Cb-TPA)^+^. This difference between I^–^/I_3_^–^ and Co^2+^/Co^3+^ electrolytes arises from the faster regeneration rate constant of Por^+^–(Cb-TPA) in the Co^2+^/Co^3+^ electrolyte compared to the I^–^/I_3_^–^ electrolyte.

## Discussion

### Intramolecular charge transfer and reduction kinetics by injected electrons

Following the photo-excitation of the porphyrin chromophore in Por–(Cb-TPA), the excited electron is injected into TiO_2_ and the resulting hole must be initially generated on the Por part. This is because of the negligible electronic communication, due to the large dihedral angle, between the two chromophores supported by UV-vis absorption, electrochemical and resonance Raman spectroscopy measurements.^10^

Electron density diagrams obtained by DFT calculations of the oxidized radical cations (Table 2) suggest that the SOMO orbitals do permeate outside of the radical geometry, which may indicate delocalization between the Cb-TPA and Por units in the oxidized state. The UV-vis absorption spectrum of the oxidized Por–(Cb-TPA) cation, with localized electron density at the Por, Cb and TPA moieties predicted by TD-DFT calculations (Fig. S2†) matches the experimental data in Fig. 4 closely, providing some validation to the calculated molecular geometries, at least in solution.

Strong delocalization in the oxidized state is inconsistent with our experimental results of slow (up to microsecond component is observed in Fig. 6), partial and heterogeneous hole transfer kinetics within the oxidized Por–(Cb-TPA). It is also inconsistent with wavelength (and therefore hole location) dependent regeneration kinetics (Fig. 7). If the cation is strongly delocalized, we expect to see (i) very fast, on the time scale of cation radical thermalization following electron injection, redistribution of electron density throughout the entire molecule; and (ii) regeneration kinetics that is independent of the probe wavelength. The above apparent discrepancy can be explained by the very small difference between the energies of radical formation at the Por and Cb-TPA moieties calculated by DFT. Electron density diagrams replotted with twice the energy density surface in Fig. S3† (isovalue 0.04) suggests the validity of this hypothesis: the lack of significant electron density on the phenylethenyl linker as well as on the 2,4,6-trimethylphenyl unit in the oxidized state of any of the cation locales suggest the lack of delocalization between the moieties.

Fig. 4 and 5 suggest that holes are generated both on the Por and the Cb-TPA chromophores in Por–(Cb-TPA), showing that some fraction of holes are transferred to the Cb-TPA part, faster than the time resolution of the transient absorption setup. Transient absorption at 1200 nm in Fig. 6 suggests that hole transfer is extended to the microsecond time scale. This component is one of the slowest intramolecular hole transfer processes reported in the literature, for example approximately 200 times slower than reported in a conjugated triarylamine-cyclometalated ruthenium polypyridyl sensitizer at –45 °C.^8^ The transient absorption measurements were repeated using a 100 ps pulsed laser (Fig. S10†) to increase time resolution to nanoseconds. Based on the ΔOD signal increase in Fig. S10(c),† it is estimated that 40% of the holes are transferred from Por to Cb-TPA chromophore within the 10 ns and 10 microsecond time scale. Such wide time range of transfer process implies a highly heterogeneous process. More precise calculation of the hole transfer yield is not possible due to the unknown extinction coefficients of the dye cation radicals on TiO_2_.

The mechanism of hole transfer can be among adsorbed dyes on the TiO_2_ surface (intermolecular) or within the same molecule, between the chromophores (intramolecular).^8^,^15^ In order to distinguish between the two mechanisms, Por–(Cb-TPA) dye was diluted on the TiO_2_ surface either by controlling the dye uptake amount by using a dilute dye solution or by adding chenodeoxycholic acid as a co-absorber to the dye solution. The dye uptake amounts were 6 × 10^–5^ mol cm^–3^ and 3 × 10^–5^ mol cm^–3^, respectively, approximately half and one fourth that of the full dye loading conditions used throughout this manuscipt. It was expected that diluting the dye increased the distance between the dye molecules and hence blocked intermolecular hole transfer. Fig. S11† shows the absorption transient for reduced dye loading conditions. The kinetics were not influenced significantly by the lower dye concentration on the surface, suggesting the hole transfer was much more likely to be intramolecular.

The amount of transferred holes from Por to Cb-TPA moiety was estimated to be around 30% using the electrochemically generated absorption spectrum of dye cations in solution (Fig. 4). Due to possible variation of the extinction coefficients between spectro-electrochemical measurements in solution to transient absorption measurements of dye-sensitised films, a large error in the estimation of the fraction of transferred holes is expected. The rather low quantum yield of hole transfer is at least partially explained by the small energy difference between the porphyrin chromophore and the Cb-TPA donor moiety, as indicated by electrochemical measurements and DFT calculations. In fact, electrochemical measurements suggested that the lowest energy oxidation state is Por^+^–(Cb-TPA) (by less than 0.1 eV). DFT calculations suggested nearly identical energies for Por–(Cb-TPA)^+^ and Por^+^–(Cb-TPA), the former being lower in energy by only 0.006 eV. Therefore, the hole transfer based on equilibrium energetics is either slightly uphill or have close to zero driving force.

To further investigate the slow, heterogeneous and only partial hole transfer kinetics, the effect of inert electrolyte composition on the transient absorption signals of TiO_2_–Por–(Cb-TPA) is examined. Note (Table 1) that the inert Co^2+^/Co^3+^ and I^–^/I_3_^–^ electrolytes had different concentrations of LiClO_4_ (0.05 M and 0.1 M, respectively) and *t*BP (0.1 M and 0.5 M, respectively). As the LiClO_4_ and *t*BP concentrations increased, the recombination kinetics at 1200 nm has become five time slower, while at 800 nm, the recombination kinetics became faster. Furthermore, the fraction of rise signal at 1200 nm in TiO_2_–Por–(Cb-TPA) has increased in magnitude and shifted to shorter time scales (Fig. S5(e)† and 4). Previously, based on detailed electron lifetime measurements of a porphyrin dimer we suggested that *t*BP could coordinate to Zn porphyrin, slowing recombination between injected electrons and oxidised species in the electrolyte.^16^ Following the same hypothesis, although this is highly speculative, intramolecular hole transfer and injected electron – dye cation radical recombination kinetics may be influenced by altering the site energies due to various coordination states of *t*BP with the Por and Cb-TPA cation moieties. The highly dispersive rates for hole transfer between Por and Cb-TPA may also be due to various geometries of coordination of *t*BP to the Por–(Cb-TPA) dye.

It is interesting to note that when *t*BP concentration is low, the reduction kinetics of Cb-TPA part by injected electron is faster than that of Por part although the distance between TiO_2_ surface and Cb-TPA is longer (Fig. S6(e)†). We don't have a clear explanation for this result. However, similar results have been observed with a dye having longer non-conjugated linker between TiO_2_ surface and conjugated framework of dye.^17^ A postulation for such observation was the different depth of injected electrons, that is, a dye with a longer non-conjugated linker would inject electron only to the surface of TiO_2_ and thus, the back transfer to the dye would be faster.^18^ However, the different depth of electron injection cannot explain the results here because the same injection process results in the two different hole locations. Another possibility is reverse hole transfer from the Cb-TPA moiety to Por, causing faster decays at 1200 nm. This is possible for some dyes where forward hole transfer was enabled at faster time scales, but after structure relaxation and possibly *t*BP coordination, back transfer became possible at longer time scales. Further efforts are needed to elucidate these observations, but here we emphasize that the distance and free energy difference are not the only factors influencing interfacial electron transfer.

### Reduction kinetics by redox couples

Regeneration rate constants of oxidized dyes for redox couples were nearly equal for wide range of redox couple concentrations except for the case of the lowest I^–^/I_3_^–^ concentration. When the amount of adsorbed dyes was reduced, regeneration kinetics measured both at 800 nm and 1200 nm were not facilitated significantly for either Por or Por–(Cb-TPA) dyes (Fig. S12–14†). This means that the higher regeneration constant of Por^+^–Cb-TPA than that of Por^+^ was not due to the higher packing density of Por on TiO_2_, which would prevent the easy penetration of the electrolyte donor species into the dye layer. The observed results therefore cannot be simply explained by accessibility to redox active sites.

For most of the concentration range, the reduction rate was governed by collision frequency. The reduction rate constant of Por^+^–(Cb-TPA) was two times faster than that of Por^+^. Since Por^+^–(Cb-TPA) can be reduced both by donor species in the electrolytes and Cb-TPA moiety, as long as the reduction by Cb-TPA moiety can compete with the reduction by the donor species, the reduction constant of Por^+^ in Por–(Cb-TPA) can be higher than that of Por^+^ without the Cb-TPA moiety. Since the reduction by Cb-TPA moiety was observed from the nano to micro second range, the reduction constant of Por^+^ can be faster. However, since the Por^+^ reduction rate constant by Cb-TPA moiety is dispersive and only roughly 20% of Por^+^ is reduced in less than a microsecond, the facilitation of the reduction rate constant of Por^+^ in Por–(Cb-TPA) was not significant.

The rate constant of Por–(Cb-TPA)^+^ was five times faster than that of Por^+^–(Cb-TPA). This can be interpreted with more exposed surface area of molecular orbitals of Cb-TPA part to electrolytes. For Por dye, the structure is two-dimensional. So the scattering cross section of the molecules is large when donor species approach the dye along the axis, which is perpendicular to the two-dimensional surface of the porphyrin ring. On the other hand, there is a much less chance to hit the porphyrin ring if the donor species come along the two-dimensional axis. The TPA moiety has a tridimensional structure, giving higher scattering cross section for all axes, resulting higher chance of electron transfer. This hypothesis is consistent with DFT calculations showing a significant hole density on the Cb-TPA chromophore in the oxidised Por–(Cb-TPA), more prominently for the Por–(Cb-TPA)^+^. Since this is the most accessible part of the molecule on surface of TiO_2_, redox interaction is more likely to occur here. Note that the higher regeneration rate constant of Por–(Cb-TPA)^+^ is significant because the free energy difference between the redox couple and Por–(Cb-TPA)^+^ is equal or only marginally different from that of Por^+^–(Cb-TPA).

We have also studied the molecular structural effect on the reduction kinetics among metal-free organic dyes having carbazole, coumarin and TPA and found that TPA showed the fastest reduction kinetics.^19^ The observations in that study is consistent with the work presented here.

Between Co^2+^/Co^3+^ complex and I^–^/I_3_^–^ redox couples, although the polarity of the redox couples is the opposite, we found the same enhancement trend. This implies that the higher reduction rate of Cb-TPA^+^ moiety was not influenced by the electrostatic force, but more likely due to higher collision frequency as suggested above.

The high regeneration rate constant at very low I^–^/I_3_^–^ concentration might be caused by increased I^–^ concentration at the vicinity of the Cb-TPA moiety. We have proposed that dye molecules increase local I_3_^–^ concentrations due to electrostatic force caused by partial charge of dye molecules or dispersion force between dye and I_3_^–^.^20^,^21^ Since the Cb-TPA moiety does not have any atoms with large partial charges, we expect that the faster regeneration kinetics was due to the dispersion force between the Cb-TPA and I^–^. On the other hand, since these forces are weak, the effect may not be so strong in the case of bulky Co complexes.

By attaching Cb-TPA moiety to Por, reduction kinetics of the oxidised Por–(Cb-TPA) was slighly facilitated. For DSSCs application, the enhancement was not practically relevant. Using optimised redox concentrations, the dye regeneration kinetics of Por was fast enough not to be limiting the performance under 100 mW cm^–2^ white light illumination. Furthermore, the observed hole transfer was only partial due to the small energy difference between two states and the large dispersion of the transfer rate. Since the reduction rate constant of Por–(Cb-TPA)^+^ is five times higher than that of Por part, we expect that if the energy difference between the Por and Cb-TPA chromophores is increased, the hole transfer yield and rate will be enhanced, and the reduction rate contant of the dichromophoric dye (at both sites) will be enhanced, so the observed phenomenon has important implications for further enhancement of photovoltaic performance. Interestingly and in line with other reports,^7^,^8^ we could not find increased charge separate state lifetime of the injected electrons in TiO_2_ and the holes at Cb-TPA moiety. Thus, to enhance the reduction kinetics, increasing distance alone may not be sufficient (Fig. 1(a)). Our result suggests that attaching an electron donating moiety with large scattering cross section to a dye is a better strategy (Fig. 1(b)).

## Conclusions

By comparing reduction kinetics between Por and Por–(Cb-TPA) adsorbed on TiO_2_ in electrolyte solutions, attaching electron donating moiety to a sensitizer was shown to increase the reduction kinetics of oxidized molecules by redox couples. Holes generated at the Por part in Por–(Cb-TPA) were partially transferred to Cb-TPA. However, the increased distance of the separated charges did not increase the charge separated state lifetime. On the other hand, the reduction rate constant of Por–(Cb-TPA)^+^ was five times faster than that of Por^+^–(Cb-TPA). This was explained by the larger exposed surface area of Cb-TPA molecular orbitals to electrolytes enhancing collision frequency. Our finding is that one can enhance reduction kinetics of oxidized molecules without changing the optical/physical properties of the molecules by attaching tridimensionally enlarged electron donor moieties.

## Supplementary Material

Supplementary information
