# Supplementary material for: Enhancement of dye regeneration kinetics in dichromophoric porphyrin–carbazole triphenylamine dyes influenced by more exposed radical cation orbitals
Source: Chem Sci. 2016 Mar 1;7(6):3506–16. doi: 10.1039/c6sc00429f (PMC6007200; doi:10.1039/c6sc00429f)
Supplement: Supplementary file 1 [file SC-007-C6SC00429F-s001.pdf]

Electronic Supplementary Material (ESI) for Chemical Science.
This journal is © The Royal Society of Chemistry 2016

Supplementary information

Enhancement of dye regeneration kinetics in dichromophoric porphyrin - carbazole triphenylamine dyes influenced by more exposed radical cation orbitals

Long Zhao,† Pawel Wagner,† Jonathan E. Barnsley,‡ Tracey M. Clarke,† Keith C. Gordon,‡ Shogo Mori§ and Attila J. Mozer*,†

† ARC Centre of Excellence for Electromaterials Science, Intelligent Polymer Research Institute, University of Wollongong, Wollongong, NSW 2522, Australia. Email: attila@uow.edu.au; Tel: +61242981429

‡ MacDiarmid Institute for Advanced Materials and Nanotechnology, Department of Chemistry, University of Otago, Dunedin, New Zealand.

§ Division of Chemistry and Materials, Faculty of Textile Science and Technology, Shinshu University, Ueda, Nagano 386-8567, Japan

Transient absorption signal fitting

A stretched exponential equation Eq. S1 was used to fit the transient absorption signal decay since variation in the concentrations of ions and anions forged around the electrolyte/dye/TiO_2 interface.[1,2] The observed lifetime (τ_{obs}) is introduced instead of the characteristic stretched relaxation time (τ_{WW}) to better describe the dye cation decay kinetics (Eqs. S2-S5).

$$\Delta OD(t) = \Delta OD_{t=0} e^{-\left(\frac{t}{\tau_{WW}}\right)^{\beta}} \quad (1)$$

$$\tau_{obs} = \frac{\tau_{WW}}{\beta}\Gamma\left(\frac{1}{\beta}\right) \quad (2)$$

$$\Gamma\left(\frac{1}{\beta}\right) = \int_0^{\infty} u^{\frac{1}{\beta}-1} e^{-u} du \quad (3)$$

$$k_{obs} = \frac{1}{\tau_{obs}} \quad (4)$$

$$k_{reg} = (k_{obs} - k_{rec}) \times \frac{1}{[M]} \quad (5)$$

where, ΔOD is the change in optical density; $\Delta OD_{t=0}$ is the initial signal magnitude; τ_{WW} is the characteristic stretched relaxation time, s; β is the stretching parameter; $\Gamma()$ is the gamma function; τ_{obs} is the observed lifetime, s; k_{obs} is the observed rate constant, s^{-1}; k_{rec} is the observed recombination rate constant, s^{-1}; k_{reg} is the observed regeneration rate constant, M^{-1}•s^{-1}; [M] is the concentration of reduced species in the redox shuttle, M.

Eq. S6 is used to fit the transient absorption decay signal of Por at 800 nm.

$$\Delta OD = \Delta OD(Por^{+}) + \Delta OD\left(TiO_2\,(e^{-})\right)$$
$$= \Delta OD_{t=0}(Por^{+}) e^{-\left(\frac{t}{\tau_{WW}(Por^{+})}\right)^{\beta(Por^{+})}} + \Delta OD_{t=0}\left(TiO_2\,(e^{-})\right) e^{-\left(\frac{t}{\tau_{WW}(TiO_2\,(e^{-}))}\right)^{\beta\left(TiO_2\,(e^{-})\right)}} \quad (6)$$

where, $\Delta OD(Por^{+})$ is the change in optical density attributed by the porphyrin cation (Por^{+}); $\Delta OD(TiO_2\ (e^{-}))$ is the change in optical density attributed by electrons in TiO_2.

Eq. S7 is used to fit the transient absorption decay signal of Por at 1200 nm.

$$\Delta OD = \Delta OD\left(TiO_2\,(e^{-})\right)$$
$$= \Delta OD_{t=0}\left(TiO_2\,(e^{-})\right) e^{-\left(\frac{t}{\tau_{WW}(TiO_2\,(e^{-}))}\right)^{\beta\left(TiO_2\,(e^{-})\right)}} \quad (7)$$

Eq. S8 is used to fit the transient absorption decay signal of Por-(Cb-TPA) at 800 nm.

$$\Delta OD = \Delta OD\left(Por^{+} - (Cb - TPA)\right) + \Delta OD\left(TiO_2\,(e^{-})\right) - \Delta OD_{HT}\left(Por - (Cb - TPA)^{+}\right)$$

$$= \Delta OD_{t=0}(Por^{+} - (Cb-TPA))e^{-\left(\frac{t}{\tau_{WW}(Por^{+} - (Cb-TPA))}\right)^{\beta(Por^{+} - (Cb-TPA))}}$$

$$+ \Delta OD_{t=0}(TiO_2(e^{-}))e^{-\left(\frac{t}{\tau_{WW}(TiO_2(e^{-}))}\right)^{\beta(TiO_2(e^{-}))}}$$

$$- \Delta OD_{t=0}(Por - (Cb-TPA)^{+})e^{-\left(\frac{t}{\tau 1_{WW}(Por - (Cb-TPA)^{+})}\right)^{\beta 1(Por - (Cb-TPA)^{+})}} \quad (8)$$

where, $\Delta OD(Por^{+}\text{-}(Cb\text{-}TPA))$ is the change in optical density attributed by the porphyrin cation (Por^{+}-(Cb-TPA)); $\Delta OD_{HT}(Por\text{-}(Cb\text{-}TPA)^{+})$ is the change in optical density attributed by the carbazole triphenylamine cation ($Por\text{-}(Cb\text{-}TPA)^{+}$) formed via hole transfer (HT); $\tau 1_{WW}$ is the characteristic stretched relaxation time of the rise feature, s; β is the stretching parameter of the rise feature.

Eq. S9 is used to fit the transient absorption decay signal of Por-(Cb-TPA) at 1200 nm.

$$\Delta OD = \Delta OD(Por - (Cb-TPA)^{+}) + \Delta OD(TiO_2(e^{-})) - \Delta OD_{HT}(Por - (Cb-TPA)^{+})$$

$$= \Delta OD_{t=0}(Por - (Cb-TPA)^{+})e^{-\left(\frac{t}{\tau_{WW}(Por - (Cb-TPA)^{+})}\right)^{\beta(Por - (Cb-TPA)^{+})}}$$

$$+ \Delta OD_{t=0}(TiO_2(e^{-}))e^{-\left(\frac{t}{\tau_{WW}(TiO_2(e^{-}))}\right)^{\beta(TiO_2(e^{-}))}}$$

$$- \Delta OD_{t=0}(Por - (Cb-TPA)^{+})e^{-\left(\frac{t}{\tau 1_{WW}(Por - (Cb-TPA)^{+})}\right)^{\beta 1(Por - (Cb-TPA)^{+})}} \quad (9)$$

where, $\Delta OD(Por\text{-}(Cb\text{-}TPA)^{+})$ is the change in optical density attributed by the carbazole triphenylamine cation ($Por\text{-}(Cb\text{-}TPA)^{+}$). The two terms, $\Delta OD(Por\text{-}(Cb\text{-}TPA)^{+})$ and $\Delta OD_{HT}(Por\text{-}(Cb\text{-}TPA)^{+})$, have the same initial absorption magnitude $\Delta OD_{t=0}(Por\text{-}(Cb\text{-}TPA)^{+})$.

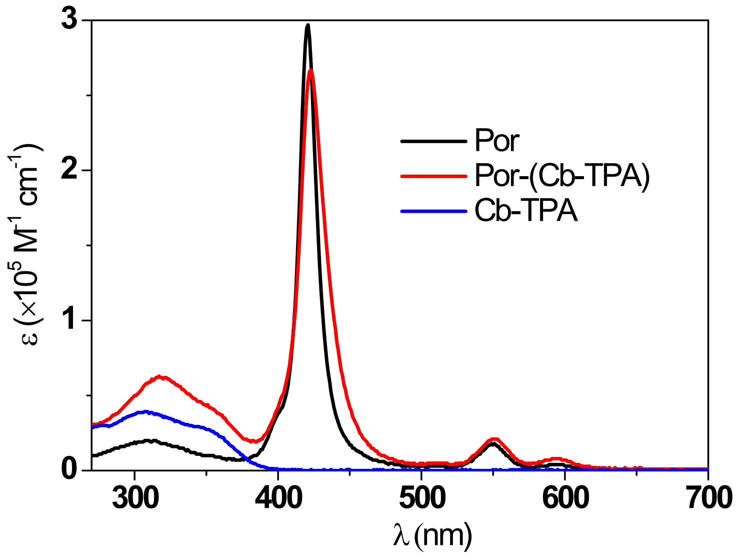

Figure S1. Molar extinction coefficient of Por, Por-(Cb-TPA) and Cb-TPA measured in dichloromethane.

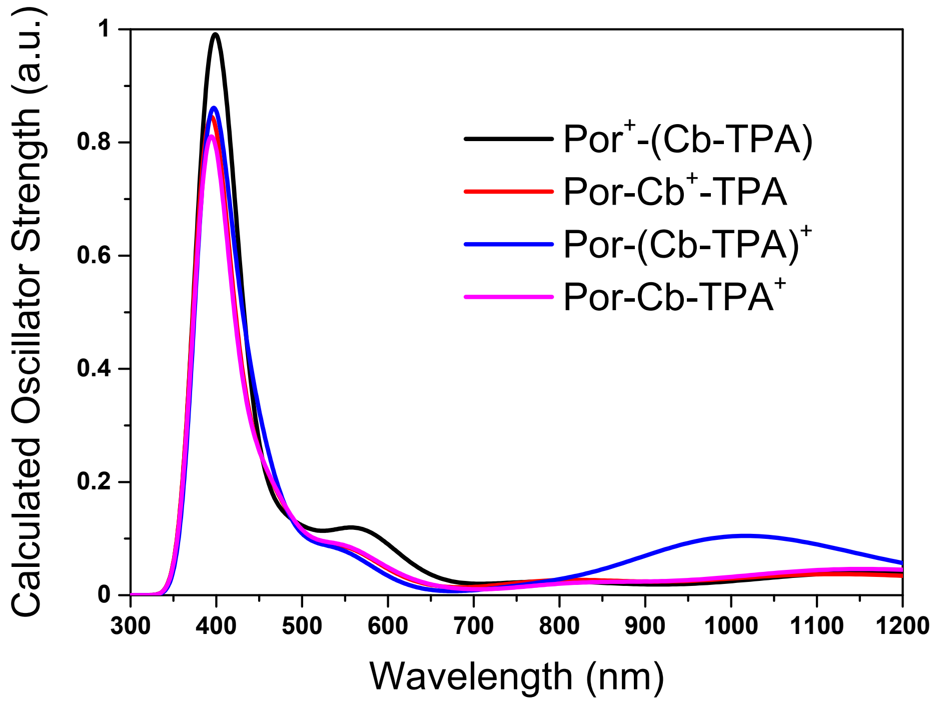

Figure S2. TD-DFT predicted UV-vis absorptions for the augmented radical species.

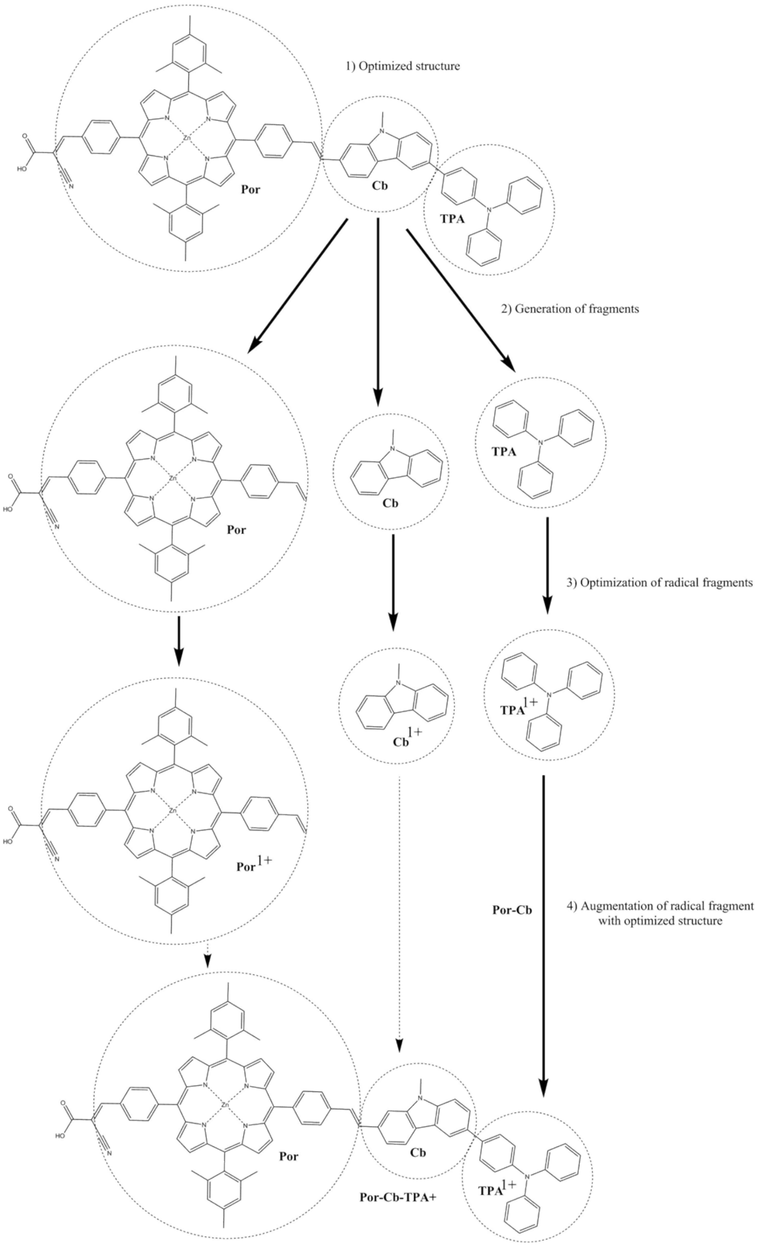

Scheme S1. A diamatic explination of the DFT calculation approach.

Occupied SOMO

Unoccupied SOMO

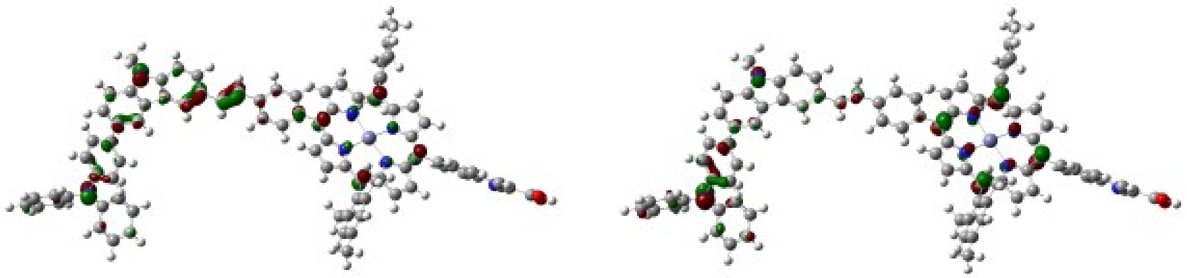

Figure S3. B3LYP calculated SOMO orbitals for Por^{+}-(Cb-TPA) at an isovalue of 0.04.

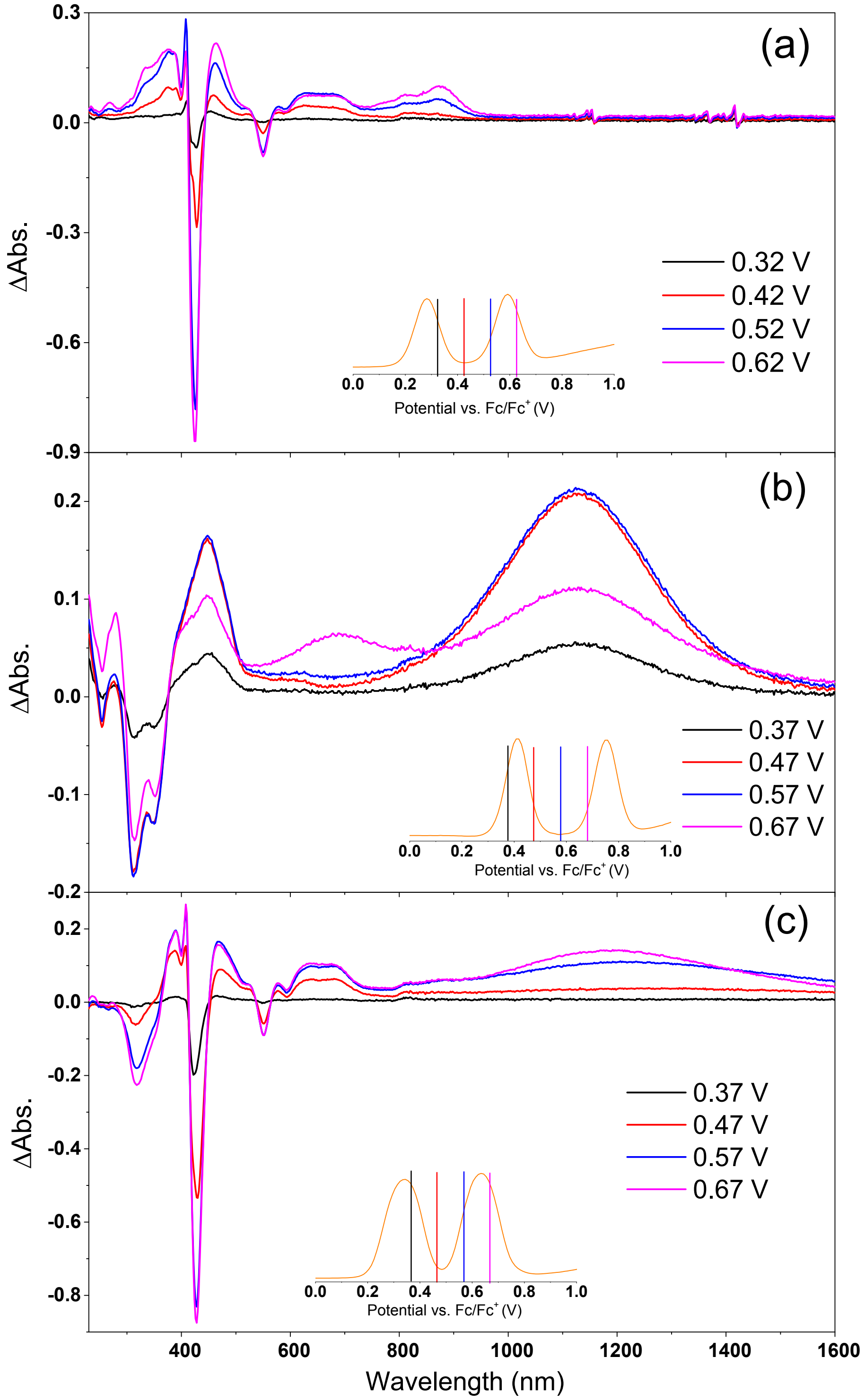

Figure S4. Spectro-electrochemical (SEC) spectra of (a) Por, (b) Cb and (c) Por-(Cb-TPA) at different oxidation potentials vs. Fc/Fc$^+$.

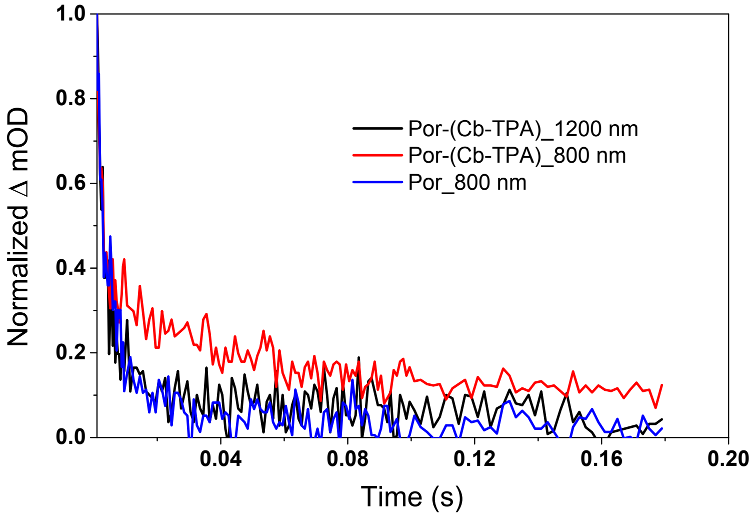

Figure S5. Transient absorption decays of TiO_2-Por-(Cb-TPA) and TiO_2-Por with the inert electrolyte I_0 on the linear scale.

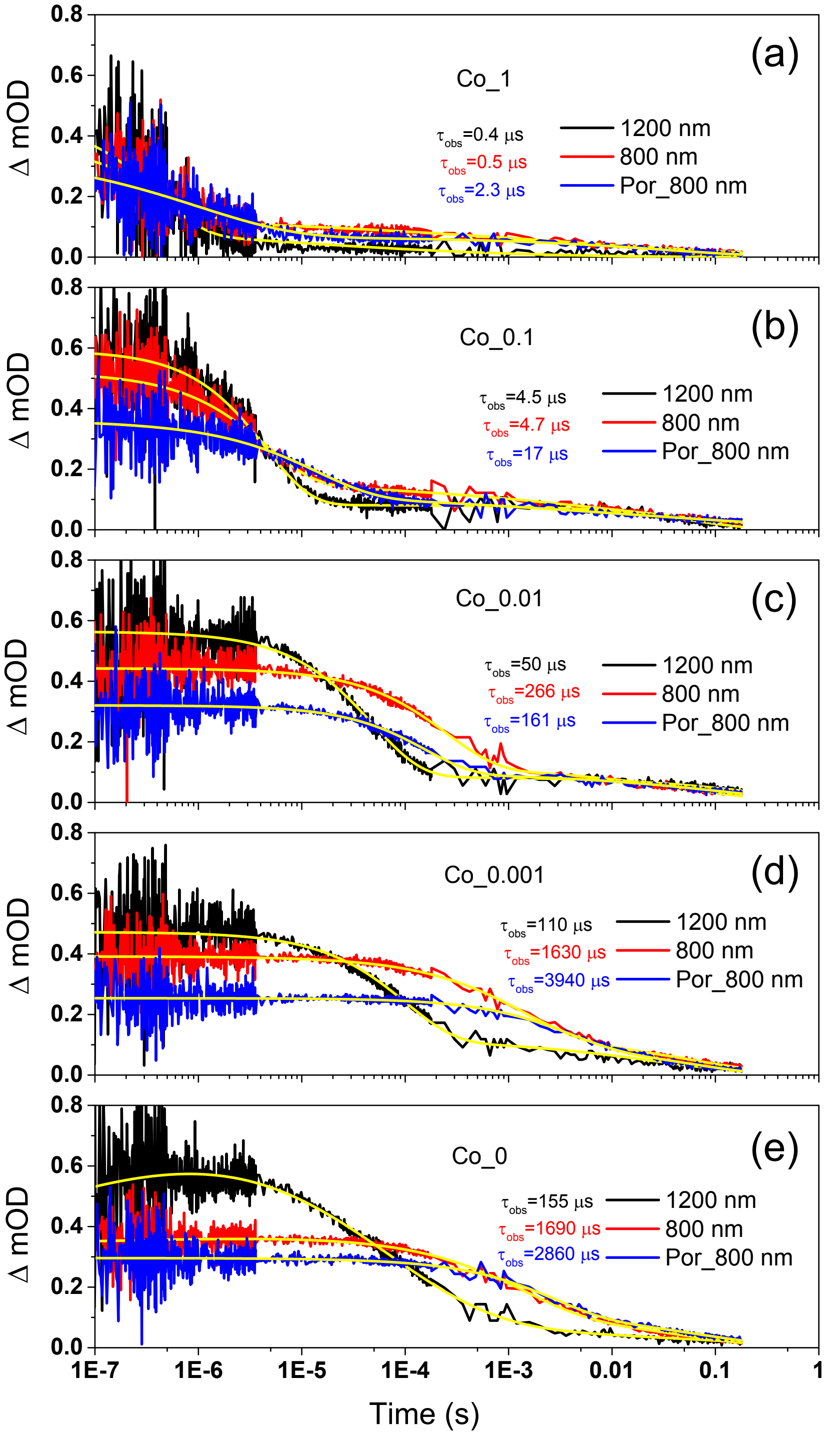

Figure S6. Transient absorption decay and fitted curves of TiO_2-Por-(Cb-TPA) probed at 1200 nm (black) and 800 nm (red) and TiO_2-Por probed at 800 nm (blue) with five Co^{2+}/Co^{3+} electrolytes after pulsed 532 nm laser irradiation. (a) Co_1, (b) Co_0.1, (c) Co_0.01, (d) Co_0.001 and (e) Co_0 (532 nm, 45-50μJ cm^{-2} $pulse^{-1}$; repetition rate 1 Hz).

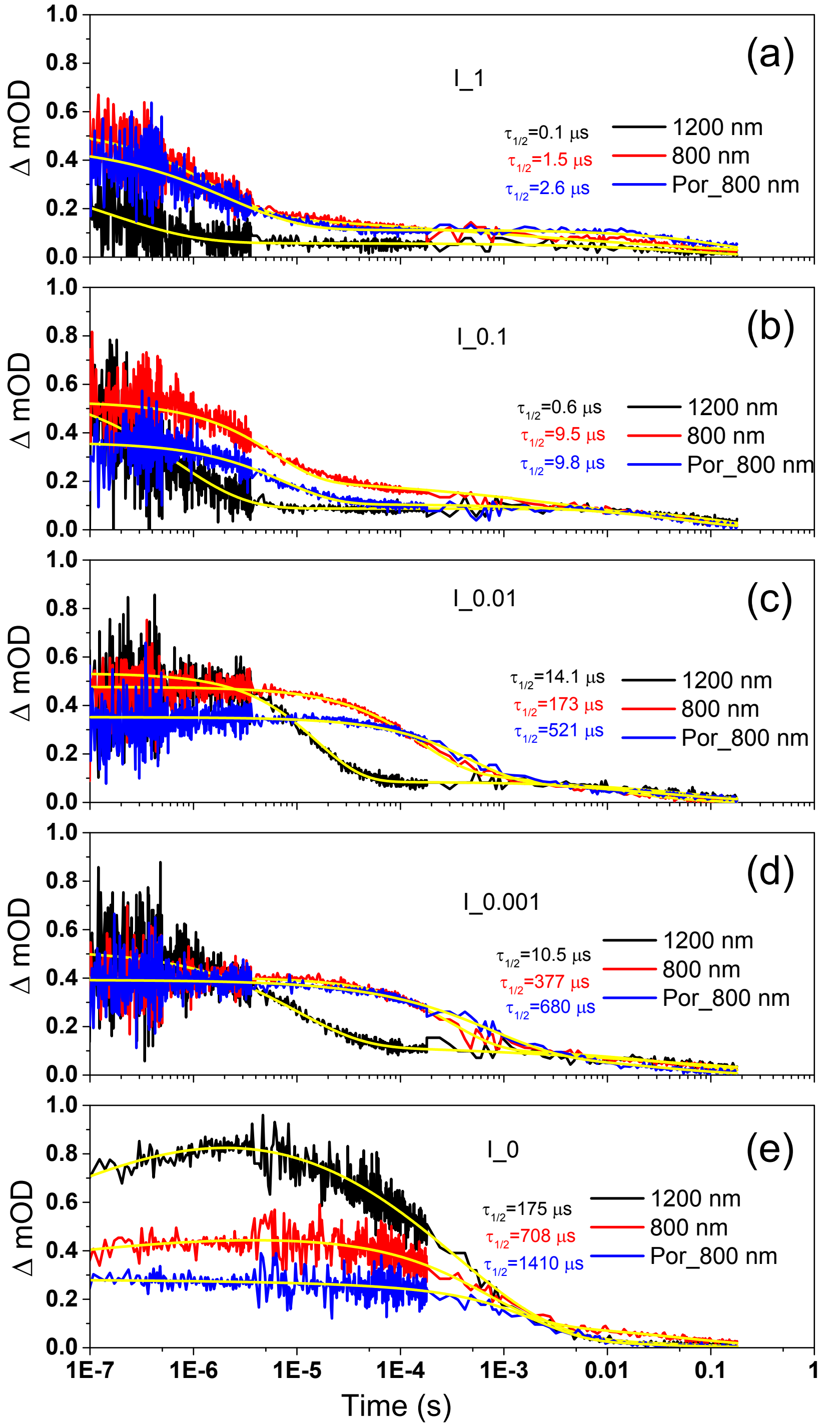

Figure S7. Transient absorption decay and fitted curves of TiO_2-Por-(Cb-TPA) probed at 1200 nm (black) and 800 nm (red) and TiO_2-Por probed at 800 nm (blue) with five I^-/I_3^- electrolytes after pulsed 532 nm laser irradiation. (a) I_1, (b) I_0.1, (c) I_0.01, (d) I_0.001 and (e) I_0 (532 nm, 45-50μJ cm^{-2} $pulse^{-1}$; repetition rate 1 Hz). Obtained signal half decay times $t_{1/2}$ are shown.

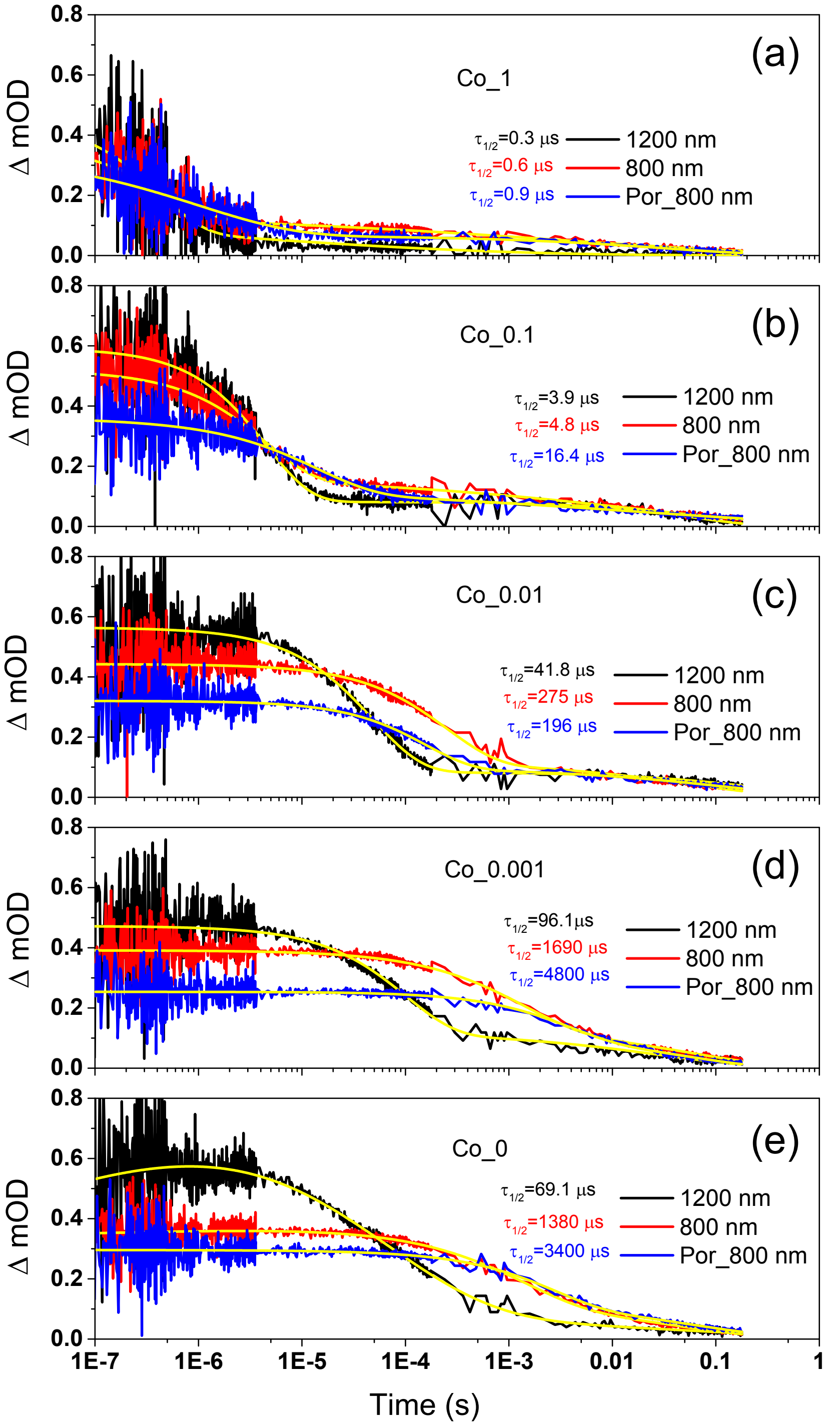

Figure S8. Transient absorption decay and fitted curves of TiO_2-Por-(Cb-TPA) probed at 1200 nm (black) and 800 nm (red) and TiO_2-Por probed at 800 nm (blue) with five Co^{2+}/Co^{3+} electrolytes after pulsed 532 nm laser irradiation. (a) Co_1, (b) Co_0.1, (c) Co_0.01, (d) Co_0.001 and (e) Co_0 (532 nm, 45-50μJ cm^{-2} $pulse^{-1}$; repetition rate 1 Hz). Obtained signal half decay times $t_{1/2}$ are shown.

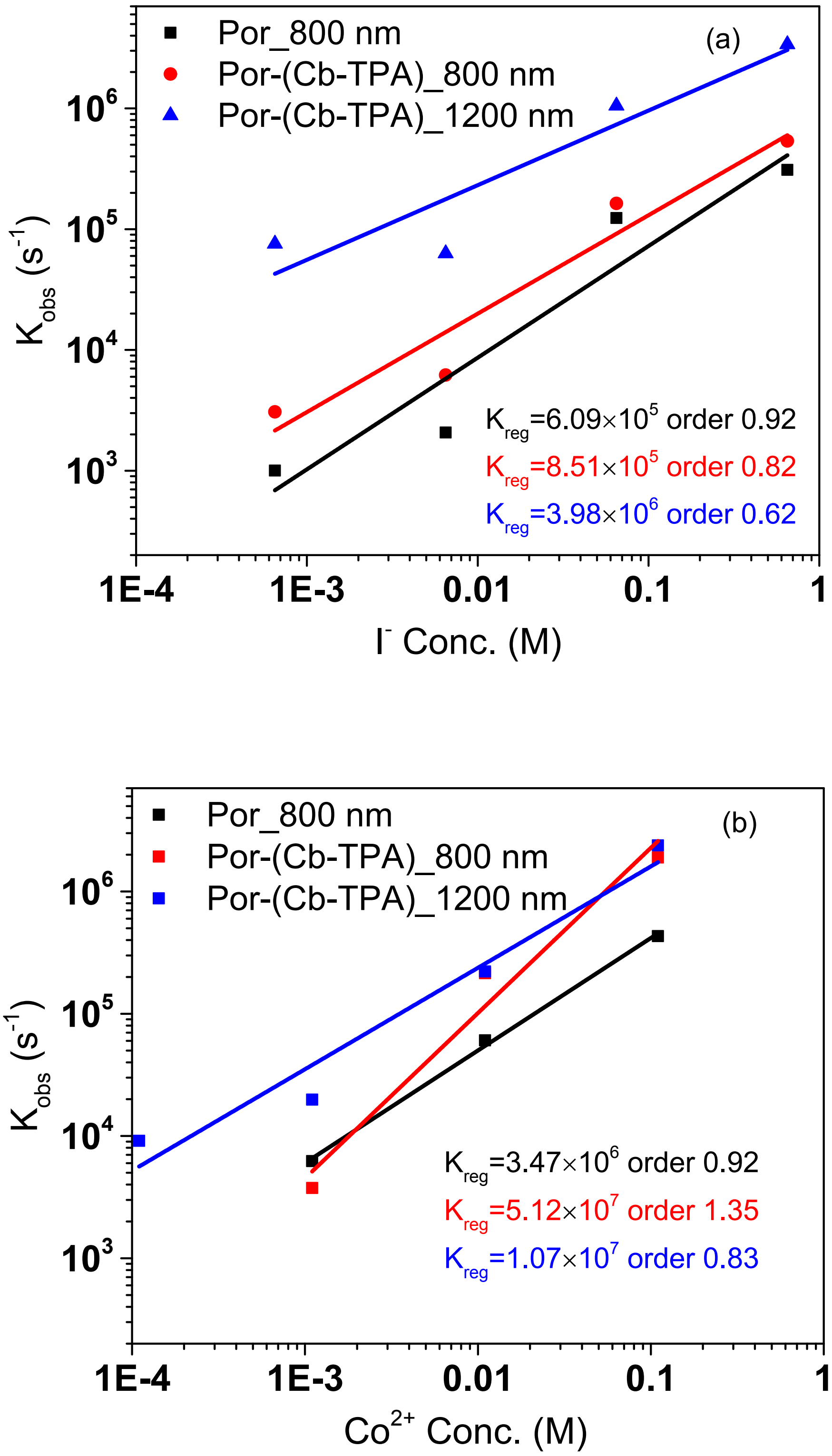

Figure S9. Observed regeneration rate of Por and Por-(Cb-TPA) versus the concentration of I^- (a) and Co^{2+} (b). Reaction order and rate constant is shown based on linear fit of the data points.

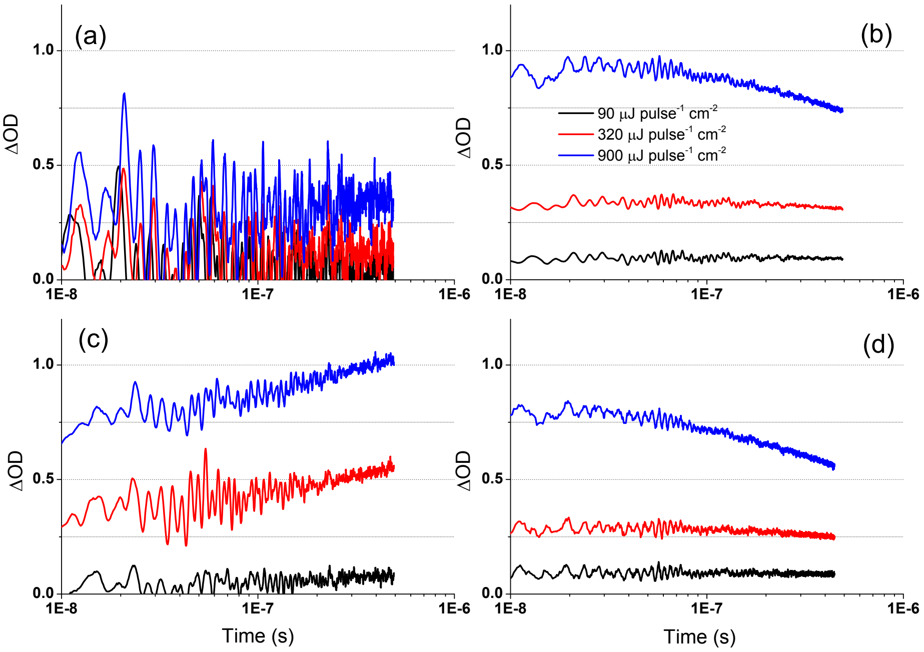

Figure S10. Transient absorption decay with different laser intensities of (a) TiO_2-Por probed at 1200 nm, (b) TiO_2-Por probed at 730 nm, (c) TiO_2-Por-(Cb-TPA) probed at 1200 nm, and (d) TiO_2-Por-(Cb-TPA) probed at 730 nm with I_0 after pulsed 532 nm laser irradiation using a 150 ps pump laser (SL230, Ekspla®). Repetition frequency: 1 Hz.

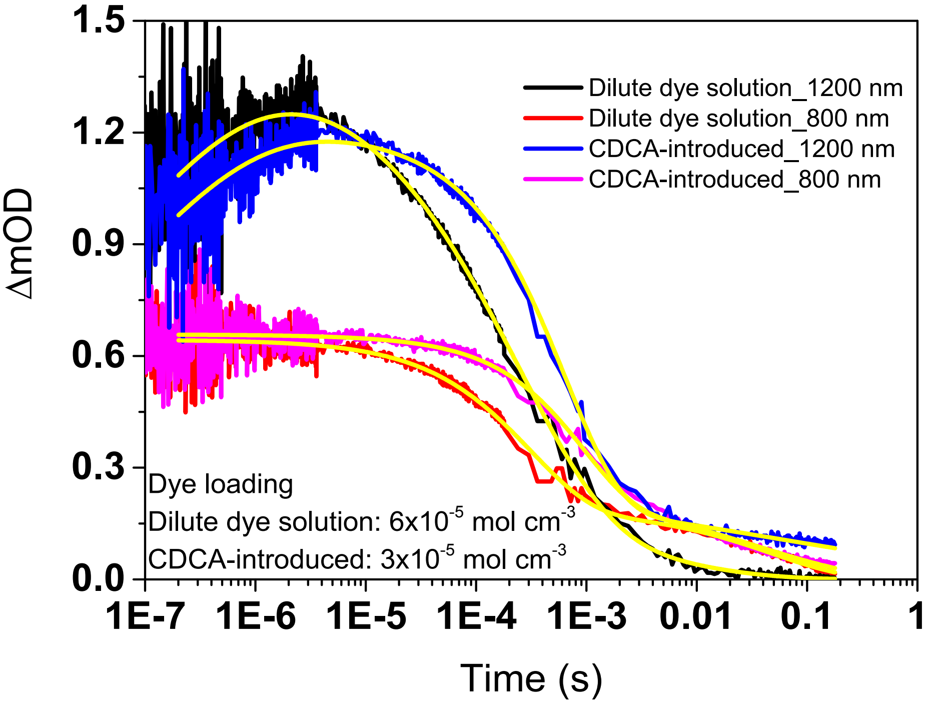

Figure S11. Transient absorption decay and fitted curves of the Por-(Cb-TPA)-sensitised TiO_2 films using dilute dye solution and CDCA-introduced conditions with the I_0 after pulsed 532 nm laser irradiation. Laser energy: 45-50 μJ cm^{-2} $pulse^{-1}$; repetition frequency: 1 Hz.

Fabrication of samples in Fig. S11: The TiO_2 layer was pasted onto 1 mm microscope glass by doctor-blading method with neither TAA coating nor $TiCl_4$ post-treatment. The TiO_2 thickness was ~10 μm after sintering under the same procedure as described in the manuscript. For dye-sensitising, 0.1 mM dye solution in THF was employed for the dilute dye solution sample while 0.1 mM dye solution with 2 mM CDCA in ethanol was employed for the CDCA-introduced sample. Dye-uptaking time was 1.5 hours.

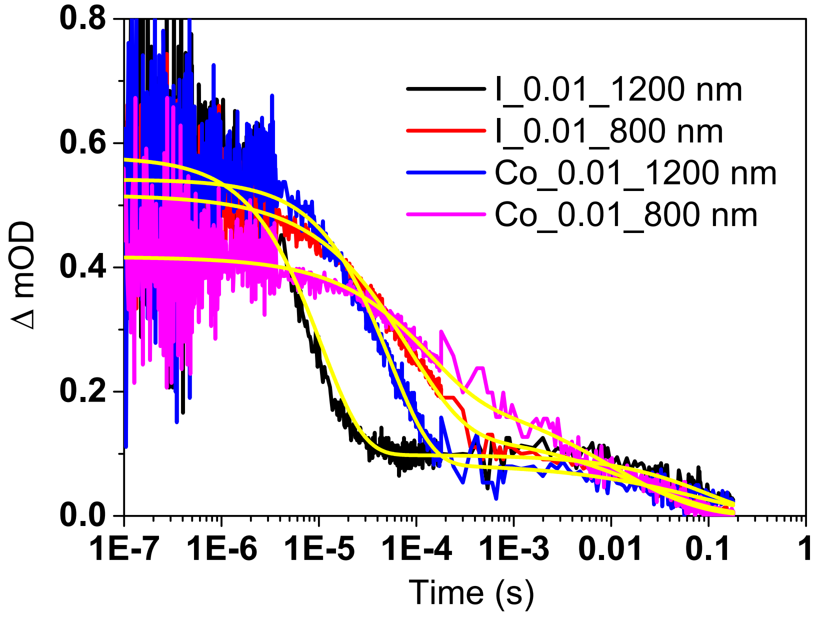

Figure S12. Transient absorption decay and fitted curves of 35% dye loaded Por-(Cb-TPA) on TiO_2 with I_0.01 and Co_0.01 after pulsed 532 nm laser irradiation. Laser energy: 45-50μJ cm^{-2} $pulse^{-1}$; repetition frequency: 1 Hz.

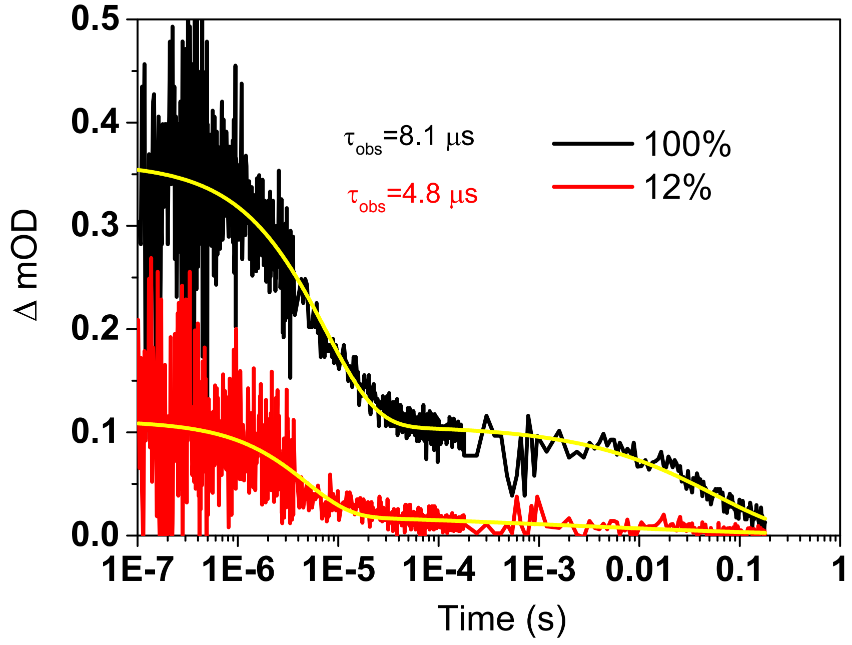

Figure S13. Transient absorption decay and fitted curves of Por with full coverage (100%) and reduced dye loading (12%) using I_0.1 after pulsed 532 nm laser irradiation probing at 800 nm. Laser energy: 45-50μJ cm^{-2} $pulse^{-1}$; repetition frequency: 1 Hz.

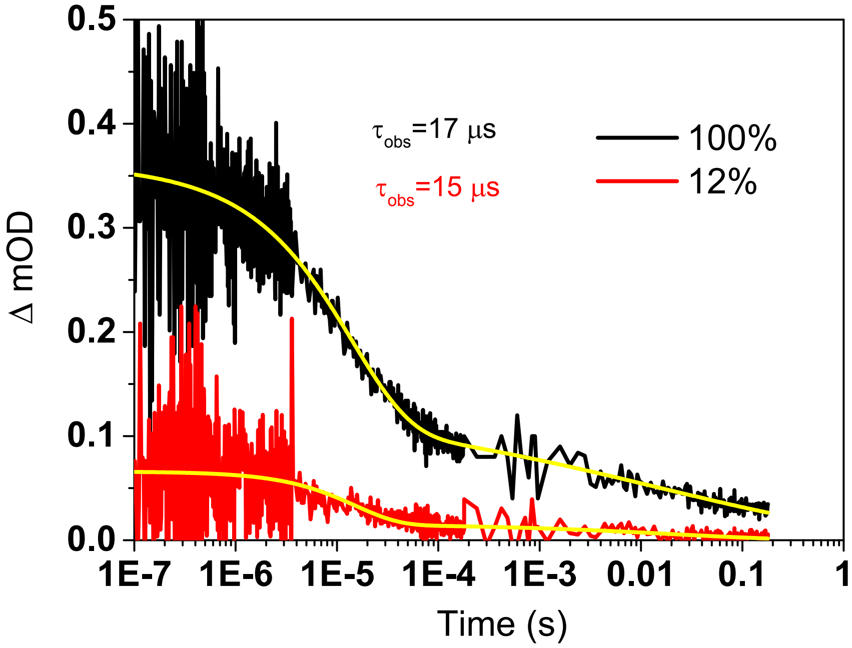

Figure S14. Transient absorption decay and fitted curves of Por with full coverage (100%) and reduced dye loading (12%) using Co_0.1 after pulsed 532 nm laser irradiation probing at 800 nm. Laser energy: 45-50μJ cm^{-2} $pulse^{-1}$; repetition frequency: 1 Hz.

Table S1. Fitting parameters for Por and Por-(Cb-TPA) using I_0 (Fig. 6)

(a) dye cation part

Sample	Wavelength (nm)	$\Delta OD\_D^{+}_{t=0}$	$\tau_{ww}\_D^{+}$ (s)	β_D^{+}	Γ_D^{+}	$\tau_{obs}\_D^{+}$ (s)	$k_{obs}\_D^{+}$ (s^{-1})
Por	800	1.90E-04	2.20E-03	0.94	0.96	2.26E-03	4.43E+02
	1200	-	-	-	-	-	-
Por-(Cb-TPA)	800	3.46E-04	6.37E-04	0.73	0.89	7.77E-04	1.29E+03
	1200	9.39E-04	3.42E-04	0.45	1.13	8.68E-04	1.15E+03

(b) electron part

Sample	Wavelength (nm)	$\Delta OD\_e_{t=0}$	τ_{ww}_e (s)	β_e	Γ_e	τ_{obs}_e (s)
Por	800	9.27E-05	3.00E-03	0.30	2.78	0.028
	1200	7.63E-05	1.10E-03	0.28	3.76	0.015
Por-(Cb-TPA)	800	1.16E-04	4.70E-02	0.44	1.15	1.23E-01
	1200	3.53E-05	6.30E-02	0.54	0.95	0.112

(c) rise part

Sample	Wavelength (nm)	$\Delta OD\_D^+_{t=0}$	$\tau 1_{ww}\_D^+$ (s)	$\beta 1\_D^+$	$\Gamma 1\_D^+$	$\tau 1_{obs}\_D^+$ (s)	$k1_{obs}\_D^+$ (s^{-1})
Por-(Cb-TPA)	800	2.19E-04	2.80E-08	0.24	7.60	8.90E-07	1.12E+06
	1200	9.39E-04	2.80E-08	0.24	7.60	8.90E-07	1.12E+06

Table S2. Fitting parameters for Por using a series of iodine- and cobalt-based electrolytes (Fig. 7 and Fig. S6).

(a) dye cation part

Electrolyte	$\Delta OD\_D^+_{t=0}$	$\tau_{ww}\_D^+$ (s)	β_D^+	Γ_D^+	$\tau_{obs}\_D^+$ (s)	$k_{obs}\_D^+$ (s^{-1})
I_1	3.52E-04	2.19E-06	0.61	0.90	3.23E-06	3.09E+05
I_0.1	2.54E-04	7.47E-06	0.85	0.92	8.09E-06	1.24E+05
I_0.01	2.50E-04	4.68E-04	0.91	0.94	4.83E-04	2.07E+03
I_0.001	2.96E-04	7.18E-04	0.64	0.89	9.98E-04	1.00E+03
I_0	1.90E-04	2.20E-03	0.94	0.96	2.26E-03	4.43E+02
Co_1	2.79E-04	9.79E-07	0.46	1.09	2.32E-06	4.31E+05
Co_0.1	2.32E-04	1.39E-05	0.75	0.89	1.65E-05	6.06E+04
Co_0.01	2.23E-04	1.57E-04	0.94	0.97	1.61E-04	6.22E+03
Co_0.001	1.54E-04	3.59E-03	0.84	0.92	3.94E-03	2.54E+02
Co_0	1.67E-04	2.61E-03	0.84	0.92	2.86E-03	3.50E+02

(b) electron part

Electrolyte	$\Delta OD\_e_{t=0}$	τ_{ww}_e (s)	β_e	Γ_e	τ_{obs}_e (s)
I_1	1.12E-04	1.73E-01	0.69	0.89	0.223
I_0.1	1.07E-04	5.73E-02	0.55	0.94	0.098
I_0.01	1.02E-04	3.17E-02	0.41	1.28	0.099
I_0.001	9.67E-05	2.51E-02	0.48	1.04	0.054
I_0	9.27E-05	3.00E-03	0.30	2.78	0.028
Co_1	6.60E-05	3.20E-02	0.42	1.21	0.091
Co_0.1	1.36E-04	1.57E-02	0.20	24.00	1.856
Co_0.01	9.72E-05	1.19E-01	0.44	1.14	0.308
Co_0.001	9.94E-05	6.00E-02	0.64	0.89	0.083
Co_0	1.29E-04	6.00E-02	0.47	1.06	0.135

Table S3. Fitting parameters for Por-(Cb-TPA) using a series of iodine- and cobalt-based electrolytes (Fig. 7 and Fig. S6).

(a) dye cation part

Electrolyte	Wavelength (nm)	$\Delta OD\_D^{+}{}_{t=0}$	$\tau_{ww}\_D^{+}$ (s)	β_D^{+}	Γ_D^{+}	$\tau_{obs}\_D^{+}$ (s)	$k_{obs}\_D^{+}$ (s^{-1})
I_1	800	3.31E-04	1.59E-06	0.77	0.90	1.86E-06	5.37E+05
	1200	3.37E-04	1.45E-07	0.50	1.01	2.95E-07	3.39E+06
I_0.1	800	3.10E-04	6.05E-06	0.96	0.98	6.13E-06	1.63E+05
	1200	5.12E-04	7.00E-07	0.65	0.89	9.49E-07	1.05E+06
I_0.01	800	3.34E-04	1.57E-04	0.93	0.96	1.62E-04	6.19E+03
	1200	4.47E-04	1.59E-05	1.00	1.00	1.59E-05	6.29E+04
I_0.001	800	2.90E-04	3.20E-04	0.95	0.97	3.26E-04	3.07E+03
	1200	3.84E-04	1.02E-05	0.68	0.89	1.32E-05	7.56E+04
I_0	800	3.46E-04	6.37E-04	0.73	0.89	7.77E-04	1.29E+03
	1200	9.39E-04	3.42E-04	0.45	1.13	8.68E-04	1.15E+03
Co_1	800	2.45E-04	4.95E-07	0.87	0.93	5.28E-07	1.90E+06
	1200	3.47E-04	4.20E-07	0.99	0.99	4.20E-07	2.38E+06
Co_0.1	800	3.57E-04	4.46E-06	0.90	0.94	4.67E-06	2.14E+05
	1200	5.11E-04	4.52E-06	1.00	1.00	4.52E-06	2.21E+05
Co_0.01	800	3.25E-04	2.50E-04	0.88	0.94	2.66E-04	3.76E+03
	1200	4.80E-04	4.83E-05	0.91	0.95	5.04E-05	1.98E+04
Co_0.001	800	2.57E-04	1.34E-03	0.73	0.89	1.63E-03	6.15E+02
	1200	3.60E-04	9.80E-05	0.81	0.91	1.10E-04	9.13E+03
Co_0	800	2.45E-04	1.30E-03	0.68	0.89	1.69E-03	5.91E+02
	1200	1.17E-03	2.78E-05	0.34	1.90	1.55E-04	6.46E+03

(b) electron part

Electrolyte	Wavelength (nm)	$\Delta OD\_e_{t=0}$	τ_{ww}_e (s)	β_e	Γ_e	τ_{obs}_e (s)
I_1	800	2.50E-04	1.16E-03	0.15	389.00	3.008
	1200	5.73E-05	7.40E-02	0.55	0.94	0.127
I_0.1	800	2.24E-04	9.36E-03	0.30	2.95	0.094
	1200	8.77E-05	1.52E-01	0.78	0.90	0.175
I_0.01	800	1.43E-04	9.48E-03	0.48	1.04	0.021
	1200	8.58E-05	7.65E-02	0.57	0.92	0.123
I_0.001	800	1.12E-04	6.48E-02	0.50	1.00	0.130
	1200	1.34E-04	5.04E-02	0.26	4.63	0.894
I_0	800	1.16E-04	4.70E-02	0.44	1.15	1.23E-01
	1200	3.53E-05	6.30E-02	0.54	0.95	0.112
Co_1	800	1.41E-04	3.05E-03	0.20	29.16	0.456
	1200	1.87E-04	1.10E-06	0.15	389.00	0.003
Co_0.1	800	1.67E-04	1.42E-02	0.26	4.63	0.249
	1200	8.14E-05	6.15E-02	0.71	0.89	0.078
Co_0.01	800	1.18E-04	5.73E-02	0.48	1.04	0.124
	1200	8.40E-05	2.83E-01	0.56	0.93	0.470
Co_0.001	800	1.35E-04	6.00E-02	0.51	0.98	0.115
	1200	1.26E-04	2.30E-02	0.38	1.50	0.092
Co_0	800	1.28E-04	3.70E-02	0.46	1.09	0.088
	1200	1.05E-04	1.52E-02	0.21	16.87	1.210

(c) rise part

Electrolyte	Wavelength (nm)	$\Delta OD\_D^{+}_{t=0}$	$\tau1_{ww}\_D^{+}$ (s)	β1_ D^{+}	Γ1_ D^{+}	$\tau1_{obs}\_D^{+}$ (s)	$k1_{obs}\_D^{+}$ (s^{-1})
I_0	800	2.19E-04	2.80E-08	0.24	7.60	8.90E-07	1.12E+06
	1200	9.39E-04	2.80E-08	0.24	7.60	8.90E-07	1.12E+06
Co_0	800	4.90E-05	1.99E-07	0.21	15.32	1.43E-05	6.99E+04
	1200	8.24E-04	1.99E-07	0.21	15.32	1.43E-05	6.99E+04

Table S4. Fitting parameters for dilute dye solution and CDCA-introduced Por-(Cb-TPA)-sensitised TiO_2 film using I_0 (Fig. S11)

(a) dye cation part

Sample	Wavelength (nm)	$\Delta OD\_D^{+}_{t=0}$	$\tau_{ww}\_D^{+}$ (s)	β_D+	Γ_D+	$\tau_{obs}\_D^{+}$ (s)	$k_{obs}\_D^{+}$ (s^{-1})
Dilute dye solution	800	4.57E-04	3.08E-04	0.77	0.89	3.58E-04	2.80E+03
CDCA-introduced		4.21E-04	9.30E-04	0.93	0.96	9.60E-04	1.04E+03
Dilute dye solution	1200	1.15E-03	2.32E-04	0.52	0.97	4.33E-04	2.31E+03
CDCA-introduced		9.50E-04	6.42E-04	0.86	0.93	6.92E-04	1.45E+03

(b) electron part

Sample	Wavelength (nm)	$\Delta OD\_e_{t=0}$	τ_{ww}_e (s)	β_e	Γ_e	τ_{obs}_e (s)
Dilute dye solution	800	1.87E-04	4.90E-02	0.64	0.89	0.068
CDCA-introduced		2.38E-04	3.70E-02	0.46	1.09	0.088
Dilute dye solution	1200	2.72E-04	1.43E-03	0.34	1.90	0.01
CDCA-introduced		2.91E-04	6.00E-02	0.20	24.00	7.20

(c) rise part (TAS at 800 nm did not consider a rise component)

Sample	Wavelength (nm)	$\Delta OD\_D^{+}_{t=0}$	$\tau 1_{ww}\_D^{+}$ (s)	β1_D+	Γ1_D+	$\tau 1_{obs}\_D^{+}$ (s)	$k1_{obs}\_D^{+}$ (s^{-1})
Dilute dye solution	1200	1.15E-03	8.50E-08	0.35	1.76	4.27E-07	2.34E+06
CDCA-introduced		9.50E-04	8.50E-08	0.37	1.51	3.47E-07	9.50E-04

Table S5. Fitting parameters for 35% dye loaded Por-(Cb-TPA) using I_0.01 and Co_0.01 (Fig. S12)

(a) dye cation part

Electrolyte	Wavelength (nm)	$\Delta OD\_D^+_{t=0}$	$\tau_{ww}\_D^+$ (s)	β_D^+	Γ_D^+	$\tau_{obs}\_D^+$ (s)	$k_{obs}\_D^+$ (s^{-1})
I_0.01	800	3.78E-04	8.33E-05	0.75	0.89	9.88E-05	1.01E+04
	1200	4.80E-04	1.06E-05	1.00	1.00	1.06E-05	9.43E+04
Co_0.01	800	2.03E-04	1.25E-04	0.80	0.91	1.42E-04	7.03E+03
	1200	4.56E-04	5.36E-05	1.00	1.00	5.36E-05	1.87E+04

(b) electron part

Electrolyte	Wavelength (nm)	$\Delta OD\_e_{t=0}$	τ_{ww}_e (s)	β_e	Γ_e	τ_{obs}_e (s)
I_0.01	800	1.39E-04	2.10E-02	0.52	0.97	3.92E-02
	1200	9.83E-05	9.60E-02	0.74	0.89	1.15E-01
Co_0.01	800	2.15E-04	1.04E-02	0.49	1.02	2.16E-02
	1200	8.45E-05	7.35E-02	0.53	0.97	1.35E-01

Table S6. Fitting parameters for Por with 100% and 12% dye amount on TiO_2 using I_0.1 and Co_0.1 probed at 800 nm (Fig. S13 and Fig. S14).

(a) dye cation part

Electrolyte	$\Delta OD\_D^+_{t=0}$	$\tau_{ww}\_D^+$ (s)	β_D^+	Γ_D^+	$\tau_{obs}\_D^+$ (s)	$k_{obs}\_D^+$ (s^{-1})
I_0.1	8.84E-05	4.83E-06	0.99	0.99	4.83E-06	2.07E+05
Co_0.1	5.03E-05	1.52E-05	1.00	1.00	1.52E-05	6.58E+04

(b) electron part

Electrolyte	$\Delta OD\_e_{t=0}$	τ_{ww}_e (s)	β_e	Γ_e	τ_{obs}_e (s)
I_0.1	2.57E-05	2.45E-03	0.19	35.94	0.463
Co_0.1	1.57E-05	2.13E-02	0.38	1.46	0.082

References:

1. A. Y. Anderson, P. R. F. Barnes, J. R. Durrant and B. C. O'Regan, *J. Phys. Chem. C*, 2011, 115, 2439-2447.
2. T. Daeneke, A. J. Mozer, Y. Uemura, S. Makuta, M. Fekete, Y. Tachibana, N. Koumura, U. Bach and L. Spiccia, *J. Am. Chem. Soc.*, 2012, 134, 16925-16928.
